# ﻿Genetic and morphometric analyses of historical type specimens clarify the taxonomy of the Ethiopian *Leptopelisgramineus* species complex (Anura, Arthroleptidae)

**DOI:** 10.3897/zookeys.1128.82176

**Published:** 2022-11-08

**Authors:** Sandra Goutte, Jacobo Reyes-Velasco, Abeje Kassie, Stéphane Boissinot

**Affiliations:** 1 New York University Abu Dhabi, Saadiyat Island, Abu Dhabi, United Arab Emirates New York University Abu Dhabi Abu Dhabi United Arab Emirates; 2 Entorno Biotico A.C., Colima, Colima, Mexico Entorno Biotico A.C. Colima Mexico; 3 Ethiopian Biodiversity Institute, Addis Ababa, Ethiopia Ethiopian Biodiversity Institute Addis Ababa Ethiopia; 4 Addis Ababa University, Addis Ababa, Ethiopia Addis Ababa University Addis Ababa Ethiopia

**Keywords:** African treefrogs, Afromontane, historical DNA, integrative taxonomy, *Leptopelisshebellensis* sp. nov., *Leptopelisxeniae* sp. nov., museomics, new species

## Abstract

Frogs of the genus *Leptopelis* have diversified in the Ethiopian Highlands to occupy forests and montane grasslands both east and west of the Great Rift Valley. Genetic studies revealed that the endemic species *Leptopelisgramineus* (Boulenger, 1898) comprises multiple unnamed taxa. A careful examination of historical type specimens is, however, needed to fully resolve the taxonomy of the group. Here we use mitochondrial DNA and morphological analyses on a large sample of recently-collected Ethiopian *Leptopelis*, as well as century-old type specimens to demonstrate that the recently resurrected *L.montanus* Tiutenko & Zinenko, 2021 (previously *Pseudocassinaocellata* Ahl, 1924) is a junior synonym of *L.rugosus* (Ahl, 1924) and corresponds to the taxon found west of the Great Rift Valley, not east as previously thought. Our results show that populations inhabiting the mountains and plateaus east of the Rift constitute a distinct and undescribed species. We provide a re-description of *L.rugosus* and describe two new species inhabiting the Highlands east of the Great Rift Valley. We provide an identification key, as well as a description of the calls of the members of the *Leptopelisgramineus* species complex.

## ﻿Introduction

*Leptopelis* is a genus of sub-Saharan treefrogs, currently counting 54 species (Frost 2021). Members of this genus occupy a great diversity of habitats, from dry savannah to swamp forest and from lowland rainforest to montane grassland. Some species have abandoned the arboreal lifestyle of their ancestors and adopted a terrestrial or semi-fossorial life. In the Ethiopian Highlands, the genus *Leptopelis* has diversified into at least seven endemic species: four fully arboreal forms (*L.vannutellii* (Boulenger, 1898), *L.ragazzi* (Boulenger, 1898), *L.susanae* Largen, 1977 and *L.yaldeni* Largen, 1977), two burrowing forms (*L.gramineus* (Boulenger, 1898) and *L.montanus* Tiutenko & Zinenko, 2021) and one species found either on low vegetation, ground or in shallow burrows (*L.diffidens* Tiutenko & Zinenko, 2021).

**Figure 1.**
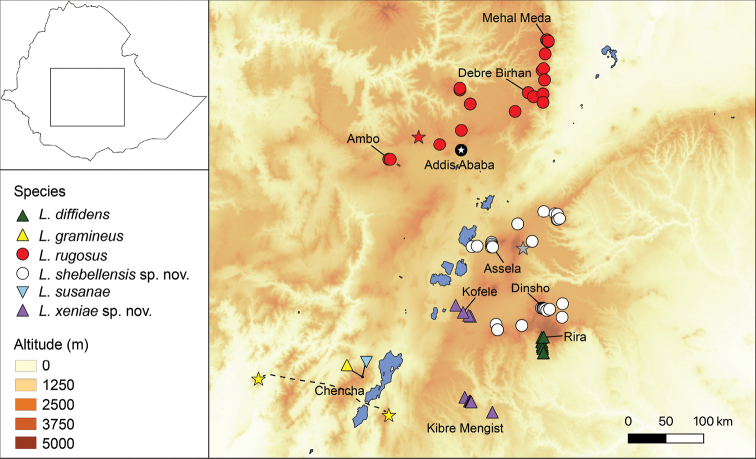
Distribution ranges of six species of the *Leptopelisgramineus* species complex. Small, more arboreal forms are represented by green (*L.diffidens*), yellow (*L.gramineus*) and purple (*L.xeniae* sp. nov.) triangles. Larger, semi-fossorial forms are represented by red (*L.rugosus)* and white (*L.shebellensis* sp. nov.) circles. The large arboreal *Leptopelissusanae* is represented by a light blue triangle. Stars indicate type localities given in the original descriptions of *L.gramineus* (yellow; dashed line between Badditù and Dimé), *Pseudocassinarugosa* (red) and *Pseudocassinaocellata* (grey), both synonymised here with *L.rugosus* (see the discussion in the main text).

The Ethiopian burrowing treefrog *Leptopelisgramineus* was described as *Megalixalusgramineus* by Boulenger (1898), based on several individuals collected in southeast Ethiopia (“Between Badditù and Dimé”, see Fig. 1) during Captain Bottego’s expedition in 1895–1897. Two species, *Pseudocassinaocellata* and *Pseudocassinarugosa*, were subsequently described by Ahl (1924), based on individuals collected further north, during Oscar Neumann’s and Carlo von Erlanger’s expedition in 1900, but later synonymised with *Leptopelisgramineus* by Largen (1977). Largen also provided a distribution map of *L.gramineus*, which spanned habitats from 1,900 m to 3,900 m a.s.l. across the Ethiopian Highlands, both east and west of the Great Rift Valley (GRV). Using mitochondrial sequences and a small number of nuclear genes, Freilich et al. (2016) showed that *L.gramineus* was likely a species complex consisting of four taxa: one occupying the highlands west of the GRV (“West” clade in Freilich et al. 2016), one occupying the plateaus and mountains east of the GRV (“Arsi” clade) and two limited to the mountain forests of the southeast (the “Kibre Mengist” and the “Katcha” clades). Using a combination of mitochondrial sequences, nuclear sequences and ddRAD-sequencing data, Reyes-Velasco et al. (2018) confirmed the presence of the four clades of Freilich et al. (2016), but also detected additional population structures east and west of the GRV. Recently, Tiutenko and Zinenko (2021) restricted the use of the name *L.gramineus* to populations of the Gamo Goffa area (i.e. west of the GRV), based on the careful examination of the collection diary and described *L.diffidens*, which corresponds to the “Katcha” lineage of Freilich et al. (2016) and to the “Harenna” lineage of Reyes-Velasco et al. (2018). In the same article, they removed the name *Leptopelisocellatus* (= *Pseudocassinaocellata*) from the synonymy with *L.gramineus*, based on the genetic results of Reyes-Velasco et al. (2018) and a few specimens collected near the type locality and applied it to the mountain and plateau populations found east of the GRV (“Arsi” clade of Freilich et al. 2016 and “Bale Mountains” clade of Reyes-Velasco et al. 2018). As a species named *Leptopelisocellatus* had been previously described from west Africa, Tiutenko and Zinenko assigned a new name to the species, *Leptopelismontanus* Tiutenko & Zinenko, 2021. These nomenclatural acts were, however, not supported by the examination of any of the historical type specimens of *Leptopelisgramineus*, *Pseudocassinaocellata* or *P.rugosa*, but based only on their respective type localities. Finally, Tiutenko and Zinenko (2021) suggested the existence of two additional species, one found on the plateaus west of the GRV (*L.* sp. “Shewa”, corresponding to the “Northern” clade of Reyes-Velasco et al. 2018), which would correspond to *L.rugosus* (= *P.rugosa*) and one from the forests of the southeast (*L. sp*. “Borana/Sidamo”, corresponding to the “Kibre Mengist” clade of Freilich et al. 2016 and Reyes-Velasco et al. 2018), but did not name or describe either of them due to a lack of data.

Here, we use mitochondrial DNA and morphological data of individuals of the *Leptopelisgramineus* complex, including the holotypes of *L.gramineus* and *P.rugosa* and the lectotype of *Pseudocassinaocellata* to clarify the taxonomic status of multiple species and populations from the Ethiopian Highlands. Our results show that *Pseudocassinarugosa* and *P.ocellata* are conspecific and distinct from *L.gramineus*. Furthermore, the lectotype of *P.ocellata* is not conspecific with the population occurring in the Bale Mountains and considered as *L.montanus* (= *P.ocellata*) by Tiutenko and Zinenko (2021). The Bale Mountains lineage thus constitutes a new species, which we describe hereafter. We also describe a forest species and resurrect *Leptopelisrugosus* (= *P.rugosa*) from the synonymy with *L.gramineus* and provide a re-description of the species.

## ﻿Materials and methods

### ﻿Sampling

Methods of sampling are discussed in detail in Reyes-Velasco et al. (2018). In brief, we collected individuals of the *Leptopelisgramineus* species complex from the Highlands of Ethiopia between 2011 and 2018 (Fig. 1; Suppl. material 5: table S1). Our study was approved by the relevant Institutional Animal Care and Use Committee at Queens College and New York University School of Medicine (IACUC; Animal Welfare Assurance Number A32721–01 and laboratory animal protocol 19–0003). Frogs were sampled according to permits DA31/305/05, DA5/442/13, DA31/454/07, DA31/192/2010, DA31/230/2010, DA31/7/2011 and DA31/02/11 provided by the Ethiopian Wildlife Conservation Authority. We photographed individuals in life and euthanised them by ventral application of 20% benzocaine gel. We extracted tissue samples and stored them in RNAlater or 95% ethanol. Adult individuals were fixed in 10% formalin for 24 to 48 hours, rinsed in water and transferred to 70% ethanol. We took additional photographs of all individuals after preservation. All specimens were deposited at the Zoological Natural History Museum of Addis Ababa University (**ZNHM**), Ethiopia. Tissue samples are deposited at the Vertebrate Tissue Collection, New York University Abu Dhabi (**NYUAD**).

### ﻿DNA extraction and sequencing of type specimens

We obtained the authorisation from the Museum für Naturkunde Berlin (ZMB) to sample a small amount of muscle or liver tissue from the holotype of *P.rugosa* (ZMB-26915) and one of the two syntypes of *Pseudocassinaocellata*, which we here formally designate as lectotype (ZMB-26913; see species account below). Tissue sampling did not result in any major visible damage to the vouchers. We did not obtain tissue samples for the lectotype of *Leptopelisgramineus* (Genoa-28564). The types specimens had most likely been fixed in formalin or in spirit, which renders the extraction of DNA challenging and requires a different protocol than when using fresh tissue. We followed the DNA extraction protocol for formalin-preserved specimens, described by Shedlock et al. (1997) and modified in Reyes-Velasco et al. (2021). A standard potassium acetate DNA precipitation protocol was then followed. We used only new reagents and conducted all DNA extractions in a marine biology lab that does not work with amphibian samples. We used multiple negative controls during every step of the DNA extraction process.

We used a high sensitivity kit in a Qubit fluorometer (Life Technologies) to measure DNA concentration, while DNA fragment size distribution and concentration was estimated on a Bioanalyzer 7500 high sensitivity DNA chip (Agilent, Santa Clara, CA, USA). We used a NEBNext FFPE DNA Repair Mix (New England Biolabs) to repair damaged bases prior to library preparation. Library preparation was performed with the use of a NEB library preparation kit. During library preparation we skipped the shredding step due to the fragmented nature of historical DNA. All libraries were pooled and sequenced on an Illumina NextSeq 550 (75 bp paired-end) at the Genome Core Facility of New York University Abu Dhabi, UAE. We used the FASTx Toolkit (Gordon and Hannon 2010) to remove Illumina adaptors and low quality reads with a mean Phred score below 20. The final average read length post-trimming was 63 bp (Suppl. material 5: table S2). We then checked for biased base composition towards the end of the reads with the programme FastQC, as biased base composition is a common phenomenon in historical or ancient samples which results from de-amination (Dabney et al. 2013). We did not find a biased base composition in our reads. Summary statistics describing the sequencing data are available in Suppl. material 5: table S2. All sequences are deposited in GenBank (Suppl. material 5: table S3).

### ﻿Assembly of mitochondrial genomes

Whole mitochondrial genomes of the type specimens of *Pseudocassinaocellata* and *Pseudocassinarugosa* were assembled from the Illumina reads using the programme MITObim (Hahn et al. 2013), which uses an iterative baiting method to generate mitochondrial contigs from short Illumina reads. We first used a published sequence of the mitochondrial genome of *Leptopelisvermiculatus* (Boulenger 1909; GenBank JX564875) as the reference mitogenome, with the default programme settings, except for a k-mer length of 21. We then re-ran the analysis using the resulting contigs from the first MITObim run. An additional eight mitochondrial genomes from other members of the *Leptopelisgramineus* species complex were also assembled following the same protocol (Suppl. material 5: table S3).

### ﻿Phylogenetic analysis of mtDNA

To assess the relationships of the holotypes of *Pseudocassinaocellata* and *P.rugosa* and the validity of the names, we reconstructed phylogenetic relationships within the *Leptopelisgramineus* species complex using all the mitochondrial protein-coding genes, as well as the ribosomal RNA 12s and 16s of the individuals for which we had full mitochondrial genomes. We performed alignments using MAFFT version 7 (Katoh and Standley 2013) and used Geneious v.9.1.6 (Biomatters Ltd., Auckland, NZ) to manually trim any poorly-aligned regions and to ensure that the protein-coding sequences were in the correct reading frame. We performed Maximum Likelihood (ML) analysis in the programme RAxML-HPC BlackBox with 1,000 bootstraps, implemented on the CIPRES Science Gateway server (Miller et al. 2010; Suppl. material 1: fig. S1A).

**Figure 2.**
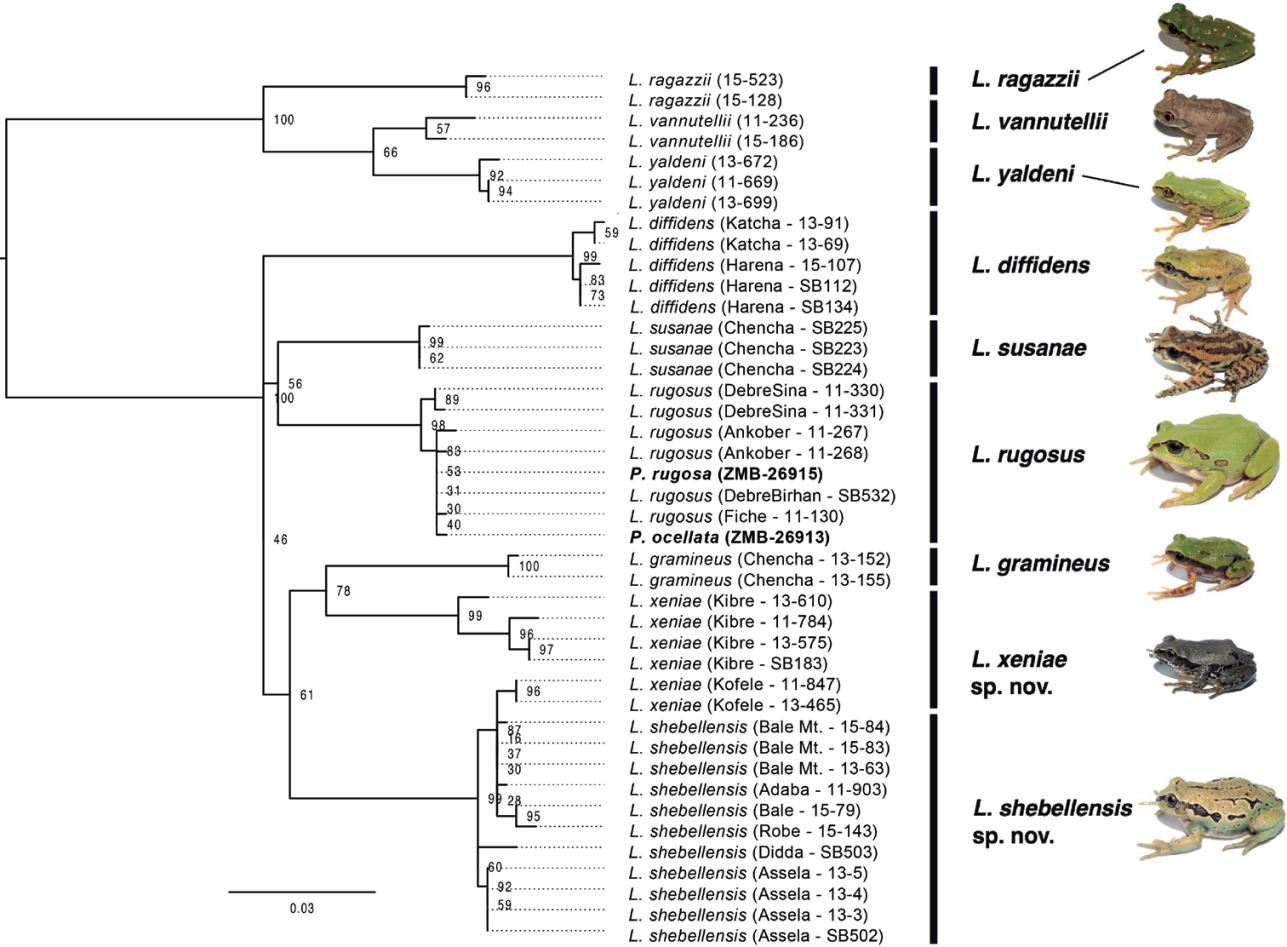
Phylogeny of the *Leptopelisgramineus* species complex. Maximum Likelihood phylogenetic inference, based on COX1. Names in bold represent historical type specimens of *Pseudocassinarugosa* (ZMB-26915) and *Pseudocassinaocellata* (ZMB-26913).

We also performed a phylogenetic analysis using only sequences of the mitochondrial protein coding gene Cytochrome c oxidase I (COX1). The reason for choosing this gene is that many more sequences of COX1 are available for Ethiopian *Leptopelis* and this gene has been shown to be informative for estimating relationships in this group of frogs (Freilich et al. 2016; Reyes-Velasco et al. 2018), thus allowing for the accurate assignment of the type specimens to species or populations. In order to test whether the phylogenetic inferences, based on COX1, only are comparable with those based on full mitochondrial genomes, we first ran a ML analysis of COX1 using only the individuals for which we have the full mitochondrial genome and compared the topologies of the two phylogenetic trees (Suppl. material 1). We then analysed the full COX1 dataset, which includes all currently-known species and population of the *Leptopelisgramineus* group and consists of 534 bp for 42 individuals, plus an outgroup (vs. 10 individuals and 13,445 bp in the full mitogenome dataset). We selected a best-fit model of nucleotide evolution with the use of the Bayesian Information Criterion (BIC) in PartitionFinder v.1.1.1 (Lanfear et al. 2012; Suppl. material 5: table S4). We performed Bayesian phylogenetic inference (BI) in MrBayes v.3.2.2 (Ronquist et al. 2012) on the CIPRES Science Gateway server (Miller et al. 2010) and additionally performed Maximum Likelihood analysis (ML) in RAxML-HPC BlackBox with 1,000 bootstraps, also implemented on the CIPRES Science Gateway server (Fig. 2, Suppl. material 2).

In certain studies of Ethiopian *Leptopelis*, species relationships were inferred using the ribosomal RNA 12s and 16s and no COX1 sequences were available for these specimens (Mengistu 2012; Tiutenko and Zinenko 2021). We thus performed an additional ML analysis on previously-published 16s sequences to establish the relationships between our samples and those collected in these studies (Suppl. material 3).

### ﻿Genetic distances

We estimated pairwise genetic distances (uncorrected P distances) of the mitochondrial data (whole mitogenome and COX1), including all codon positions, both transitions and transversions and Gamma distributed rates amongst sites in the programme MEGA X (Kumar et al. 2018). Genetic distances are presented in Suppl. material 5: tables S5, S6.

### ﻿Morphometric measurements

We measured 116 individuals that were collected in recent years, as well as type specimens of *Leptopelisgramineus* (lectotype Genoa-28564; paralectotypes Genoa-49850-1 & Genoa-49850-2), *Pseudocassinaocellata* (ZMB-26913) and *P.rugosa* (ZMB-26915) using a digital caliper (resolution ± 0.01 mm). We took 19 linear morphometric measurements for each specimen (Table 1, Suppl. material 5: table S7), which are defined in Watters et al. (2016) and were shown to be useful for morphological differentiation of anurans.

**Table 1.** Summary of the linear morphometric measurement for the *Leptopelisgramineus* species complex.

Species	sex	N	SVL	HW	HL	SL	NS	IND	EN	IOD	ETD	TD
*L.diffidens*	F	8	42 ± 5.5	15.6 ± 1.6	12.7 ± 1	5.5 ± 0.7	2.7 ± 0.5	3.3 ± 0.6	2.5 ± 0.3	3.9 ± 0.7	1.2 ± 0.3	1.8 ± 0.2
	M	20	27.5 ± 2.5	10.3 ± 1.1	9 ± 0.9	3.8 ± 0.3	2 ± 0.3	2.4 ± 0.3	1.7 ± 0.3	3.3 ± 0.4	0.7 ± 0.2	1.6 ± 0.4
*L.gramineus*	F	3	34.5 ± 8.8	13.9 ± 3.6	11.2 ± 2.5	5 ± 1.6	1.9 ± 0.9	2.5 ± 0.5	2 ± 0.6	4.2 ± 1.1	0.8 ± 0.6	1.8 ± 0.5
	M	8	30.3 ± 5.8	11.4 ± 2.7	9.5 ± 1.5	4.2 ± 0.6	2 ± 0.2	2.6 ± 0.2	1.7 ± 0.3	3.4 ± 0.8	0.7 ± 0.2	1.7 ± 0.4
*L.rugosus*	F	4	47.8 ± 5.3	16.4 ± 0.7	14.3 ± 0.7	6.7 ± 0.7	3.6 ± 0.4	4.4 ± 0.5	2.8 ± 0.4	4.3 ± 0.6	1.4 ± 0.6	2 ± 0.4
	M	22	38.7 ± 2.7	14.3 ± 1.4	11.8 ± 1.1	5.2 ± 0.4	2.9 ± 0.2	3.4 ± 0.3	2.1 ± 0.3	4.2 ± 0.5	0.9 ± 0.3	2.3 ± 0.4
*L.shebellensis* sp. nov.	F	8	53.4 ± 5.3	18.9 ± 2.3	15.8 ± 2.5	7 ± 1	3.8 ± 0.3	4.6 ± 0.5	3.3 ± 0.8	5.5 ± 0.8	1.2 ± 0.6	3.1 ± 0.5
	M	24	36.2 ± 3.3	13.2 ± 1.5	11.8 ± 1.3	5 ± 0.5	2.7 ± 0.3	3.1 ± 0.4	2.3 ± 0.3	3.8 ± 0.5	0.8 ± 0.3	2.1 ± 0.2
*L.susanae*	F	3	49.6 ± 6.6	18.1 ± 2.4	16.4 ± 2.2	7.4 ± 0.7	4.2 ± 0.2	4.6 ± 0.2	3.7 ± 0.4	5.9 ± 0.3	1.3 ± 0.4	2.6 ± 0.8
	M	12	33.2 ± 3.7	11.8 ± 1.3	11 ± 0.7	5 ± 0.4	2.6 ± 0.3	3.3 ± 0.3	2.4 ± 0.2	3.7 ± 0.3	0.9 ± 0.2	1.9 ± 0.3
*L.xeniae* sp. nov.	F	2	43.5 ± 5.7	15.9 ± 1.4	12.7 ± 0.4	5.1 ± 0.7	2.9 ± 0.4	3.2 ± 0	1.7 ± 0.3	3.3 ± 0.3	0.7 ± 0.1	2.1 ± 0.2
	M	20	27.6 ± 2.0	10.2 ± 0.6	9.1 ± 0.5	3.9 ± 0.3	2.2 ± 0.3	2.5 ± 0.3	1.7 ± 0.4	2.9 ± 0.4	0.7 ± 0.2	1.9 ± 0.3
			**ED**	**UEW**	**FLL**	**HAL**	**FinDW**	**THL**	**TL**	**FL**	**Toe4DW**	**MTL**
*L.diffidens*	F	8	4.5 ± 0.4	3 ± 0.4	9.6 ± 1.3	12.6 ± 1.9	1.5 ± 0.2	15.9 ± 3	15.6 ± 1.5	20.5 ± 2.6	1.4 ± 0.3	2.8 ± 0.5
	M	20	3.2 ± 0.4	2.5 ± 0.3	6.2 ± 0.9	8.1 ± 0.9	0.9 ± 0.1	10.9 ± 1.5	10.2 ± 0.9	12.5 ± 0.9	0.9 ± 0.1	1.8 ± 0.2
*L.gramineus*	F	3	4.2 ± 0.4	2.6 ± 0.5	8.5 ± 1.5	NaN ± NA	NaN ± NA	11.2 ± 2.1	11 ± 2.6	NaN ± NA	1.1 ± 0.2	1.8 ± 0.7
	M	8	3.6 ± 0.9	2.6 ± 0.5	6.3 ± 1.2	7.4 ± 0.5	0.8 ± 0.1	10.9 ± 1.2	10.3 ± 1.2	11.5 ± 1.2	0.9 ± 0.2	2 ± 0.7
*L.rugosus*	F	4	4.4 ± 0.3	3.5 ± 0.3	10.4 ± 1.3	13.5 ± 1	1.3 ± 0.3	20.5 ± 1.2	17.8 ± 1.4	21.3 ± 0.7	1.2 ± 0.4	3.7 ± 0.4
	M	22	4.1 ± 0.5	3.1 ± 0.2	7.8 ± 1.1	10.9 ± 0.8	1.2 ± 0.2	14 ± 1.5	13.2 ± 0.9	16.6 ± 1.3	1.1 ± 0.2	2.8 ± 0.4
*L.shebellensis* sp. nov.	F	8	5.8 ± 1.6	4.1 ± 1.1	10.8 ± 1.8	14.8 ± 1.8	1.3 ± 0.4	16.7 ± 2	16.1 ± 1.5	22.8 ± 2.5	1 ± 0.2	3.7 ± 0.6
	M	24	4.2 ± 0.7	2.8 ± 0.5	8 ± 1.2	10.2 ± 1	0.9 ± 0.2	12.7 ± 1.4	11.8 ± 1	14.8 ± 1.2	0.8 ± 0.2	2.4 ± 0.3
*L.susanae*	F	3	5.7 ± 1.3	3.9 ± 0.8	12.1 ± 1.3	16 ± 0.8	2.7 ± 0.2	22.5 ± 1	21.2 ± 2.1	24.6 ± 2.2	2.5 ± 0.3	2.9 ± 0.6
	M	12	4.2 ± 0.5	3.1 ± 0.6	7.4 ± 1.1	10.6 ± 0.8	1.8 ± 0.2	14.9 ± 0.9	14.8 ± 1	16.5 ± 1.4	1.6 ± 0.2	1.7 ± 0.2
*L.xeniae* sp. nov.	F	2	4.8 ± 0.4	3.3 ± 1.1	9.9 ± 1.8	12.8 ± 1.1	1.4 ± 0.1	16.8 ± 2.8	16 ± 3.4	20.2 ± 3.4	1.4 ± 0.1	2.4 ± 0.3
	M	20	3.3 ± 0.4	2.4 ± 0.3	6.1 ± 0.7	8.2 ± 0.7	0.8 ± 0.2	10.2 ± 1.4	9.9 ± 0.9	12 ± 1	0.8 ± 0.2	1.8 ± 0.3

List of abbreviations: **ED** eye diameter; **EN** eye-nostril distance; **ETD** eye-tympanum distance; **FinDW** longest finger disc width; **FL** foot length; **FLL** forearm length; **HAL** hand length; **HL** head length; **HW** head width; **IND** inter-nares distance; **IOD** inter-orbital distance; **MTL** metatarsal tubercle length; **NS** snout-nostril distance; **SL** snout length; **SVL** snout-vent length; **THL** thigh length; **TD** tympanum diameter; **TL** tibia length; **Toe4DW** fourth toe disc width; **UEW** upper eyelid width.

### ﻿Statistical analyses of linear morphometric measurements

**Figure 3.**
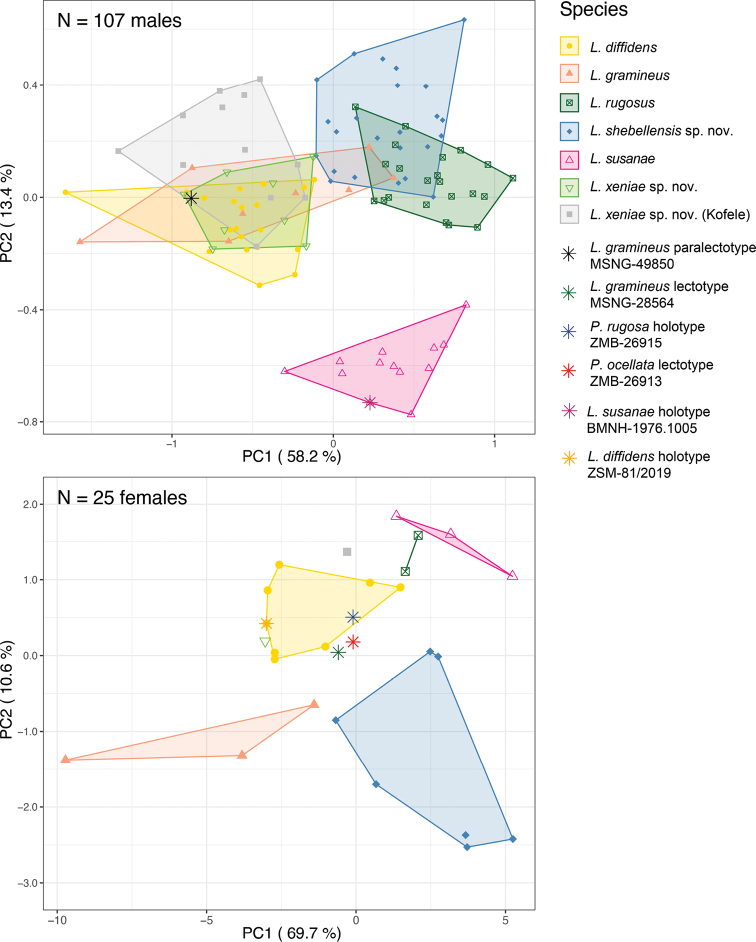
Morphometric analysis of the *Leptopelisgramineus* species complex. Scatter plots of principal component analyses, based on 16 morphometric measurements in 107 males (top) and 25 females (bottom). Type specimens of *Leptopelisgramineus*, *Pseudocassinarugosa* and *Pseudocassinaocellata* are represented by stars on the plot, but were not included in the analyses because of the important degree of shrinkage during their preservation.

We analysed males and females separately due to sexual dimorphism (Fig. 3; Table 1). We included the 18 adult individuals measured by Tiutenko and Zinenko in their recent paper (Tiutenko and Zinenko 2021) for a total sample size of 136 adults (108 males, 28 females). As sixteen measurements were shared in both datasets, we thus analysed the two datasets jointly using those measurements and excluding HAL, FL and FinDW from our dataset. We used the R package *FactoMineR* (Lê et al. 2008). As a result of shrinkage due to variable conditions of fixation and long-term preservation of type specimens, we ran discriminant analyses on recently-collected individuals only, in order to select the measurements best discriminating between species (removing the types of *Leptopelisgramineus*, *Pseudocassinarugosa* and *P.ocellata*). We then compared type specimens to the results. To determine the best discriminating morphometric measurements, we first ran a discriminant analysis and an ANOVA, followed by a Tukey HSD on each measurement selected by the discriminant analysis. Suppl. material 5: tables S8, S9 show the results of statistical analyses on linear morphometric measurements.

Morphometric measurements were all log-transformed prior to analysis in order to approach normality. To correct for body size in our measurements, we used ratios of measurements over snout-vent length. We did not use other adjustment method such as the one proposed by Lleonart et al. (2000) and used by others (e.g. Onn et al. 2018) to correct for allometric growth, because this method relies on coefficients calculated on populations and, therefore, artificially segregates individuals in a priori-determined groups. In addition, this method requires to measure multiple individuals of a given population before calculating the adjusted variables. Given that our goal here is to define characters that may be used for species identification without any a priori, we chose to resort to a size correction only based on the individual’s own mensuration.

### ﻿Recording and analysis of advertisements calls

Spontaneously calling males were located acoustically or visually between 20:00 and 02:00 h. We recorded advertisement calls in situ at a distance between 0.5–2 m to avoid near-field effects (Rossing 2007) or excessive attenuation or distortion of the sound. We used a Sennheiser ME66 directional microphone with a Sennheiser K6 powering module and an Olympus LS-100 or a Marantz PMD661 MKII recorder at a sampling rate of 44.1 kHz at 16 bits. Comments were recorded at the end of each recording using a Sennheiser ME62 microphone. The exact distance between the microphone and the calling male was measured with a Leica E7100i laser meter (precision: 3 mm) after the capture of the frog. When possible, video recordings were taken simultaneously with an infrared camcorder (SONY DCR-SR85) and custom-made Colorado Para Tech infrared lights to ensure the identity of the focal individual. Videos were subsequently used to select the focal male’s calls in recordings containing vocalisations of multiple individuals. The audio recordings are available at the Fonoteca Zoologica sound collection (https://www.fonozoo.com).

**Figure 4.**
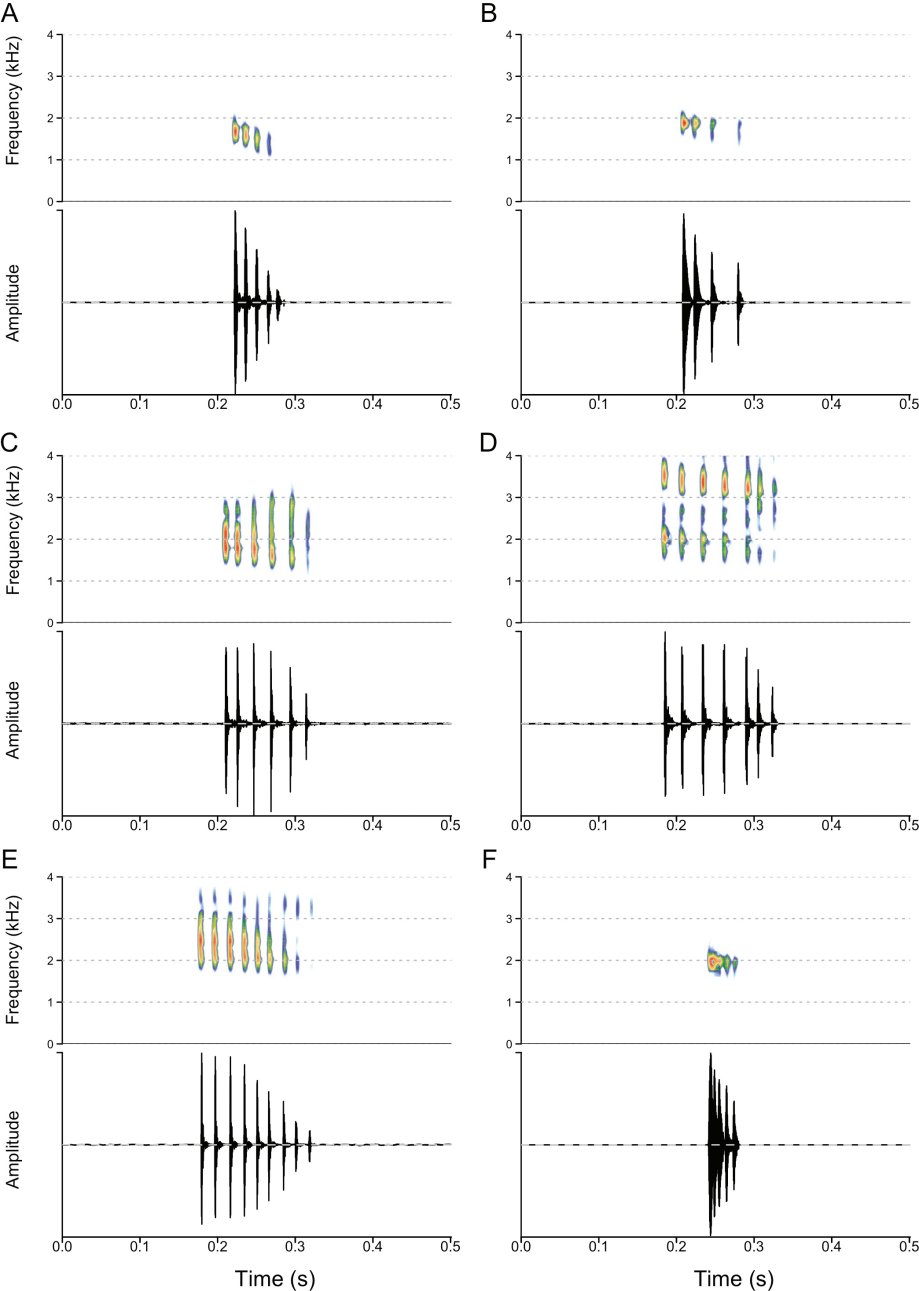
Advertisement calls of six *Leptopelis* species inhabiting the Ethiopian Highlands **A***L.shebellensis* sp. nov. (SB61) **B***L.rugosus* (SB609) **C***L.gramineus* (SB212) **D***L.xeniae* sp. nov. (SB169) **E***L.diffidens* (SB134) **F***L.susanae* (SB223).

Advertisement calls were analysed using Avisoft SAS (Sprecht 2017). We used a note-centred terminology scheme as described in Köhler et al. (2017), where the call constitutes a coherent unit and may contain one or several sub-units (notes), which, in turn, may contain distinct or indistinct pulses. We extracted six temporal and four spectral acoustic traits from our audio recordings (Table 2, Suppl. material 5: table S10): note duration, inter-call interval duration, number of pulses per note, inter-pulse interval duration, pulse rate, relative time of peak amplitude, call peak frequency, call frequency bandwidth, minimal and maximal call frequencies. Notes and pulses were labelled semi-automatically using the pulse train analysis function and subsequently by adjusting labels by eye. Sampling frequency was adjusted to 22,050 Hz. Spectral traits were extracted from the spectrogram using the automatic parameter measurement function on spectrograms using a Fast Fourier Transformation (FFT) length of 512, Hamming windowing, 50% frame size and 99.43% overlap between contiguous windows. All values were exported and averaged per individual and then per species in the R environment (R Core Team 2020; Table 2, Suppl. material 5: table S10). Spectrograms and oscillograms of the calls were plotted using the R package *seewave* (Sueur et al. 2008; Fig. 4).

**Table 2.** Summary of call acoustic characters for the *Leptopelisgramineus* species complex.

Species	N individuals	N notes	Note duration (ms)	Inter-note interval (s)	Call rate	Pulses per note	Inter-pulse duration (ms)
*L.diffidens*	8	188	130 ± 21	12.08 ± 6.18	0.10 ± 0.04	7 ± 2	22 ± 4
*L.gramineus*	2	19	93 ± 6	16.46 ± 3.17	0.07 ± 0.01	6 ± 1	19 ± 2
*L.rugosus*	6	108	65 ± 21	13.12 ± 7.47	0.10 ± 0.04	4 ± 1	20 ± 5
*L.shebellensis* sp. nov.	4	89	57 ± 5	8.28 ± 1.34	0.13 ± 0.02	5 ± 0	11 ± 2
*L.susanae*	2	50	25.6 ± 2.1	11.21 ± 3.27	0.10 ± 0.02	5.2 ± 0	11 ± 2
*L.xeniae* sp. nov.	4	51	175 ± 25	14.16 ± 6.50	0.09 ± 0.03	7 ± 1	30 ± 5
	**Pulse rate (s-1)**	**Peak frequency (Hz)**	**Min frequency (Hz)**	**Max frequency (Hz)**	**Frequency bandwidth (Hz)**	**Relative time of peak amplitude (ms)**	
*L.diffidens*	53 ± 8	1928 ± 235	1489 ± 218	2880 ± 286	1386 ± 203	39 ± 10	
*L.gramineus*	62 ± 9	1863 ± 154	1445 ± 173	2832 ± 201	1385 ± 375	21 ± 9	
*L.rugosus*	61 ± 14	1769 ± 60	1448 ± 140	2192 ± 64	742 ± 82	9 ± 5	
*L.shebellensis* sp. nov.	88 ± 3	1616 ± 265	1211 ± 253	2192 ± 132	974 ± 163	21 ± 15	
*L.susanae*	209 ± 20	1986 ± 130	1611 ± 66	2445 ± 201	832 ± 133	7 ± 6	
*L.xeniae* sp. nov.	39 ± 7	2231 ± 585	1546 ± 73	3562 ± 505	2013 ± 573	44 ± 24	

## ﻿Results

### ﻿Phylogenetic analysis of mitochondrial DNA sequences

Results of both the Bayesian Inference (BI) and Maximum Likelihood (ML) analyses of the full COX1 dataset were largely congruent with each other and with previous analyses by Freilich et al. (2016) and Reyes-Velasco et al. (2018; Fig. 2, Suppl. material 2). The arboreal species of Ethiopian *Leptopelis* (*L.ragazzii*, *L.vannutellii* & *L.yaldeni*) form a monophyletic group, which is sister to the remaining species and populations, referred hereafter as the *L.gramineus* species complex. The complex consists of six distinct lineages: (1) a lineage consisting of frogs from the Harenna forest, which correspond to *L.diffidens*; (2) a lineage corresponding to the arboreal species *L.susanae*; (3) a lineage consisting of frogs from the plateaus and mountains west of the GRV, *L.rugosus*; (4) a lineage corresponding to *L.gramineus* as defined by Tiutenko & Zinenko, 2021; (5) a lineage of frogs from the forests of the southeast corresponding to the “Kibre Mengist” clade of Freilich et al. (2016) and Reyes-Velasco et al. (2018) and (6) a lineage consisting of frogs from the plateaus and mountains east of the GRV. It should be noted that the *L.gramineus* lineage from the Chencha Highlands was mistakenly assigned to *L.susanae* in Reyes-Velasco et al. (2018). However, upon closer examination and comparison with additional material, it was determined that these individuals, in fact, represent *L.gramineus*. The individuals of *L.susanae* in the present study were carefully examined morphologically and fit the original description of Largen (1977). Despite the multiple clades being largely congruent with previous studies, the relationships amongst clades vary when compared to previous studies and many of the deeper nodes are not well supported (Suppl. material 2). These phylogenetic discordances have previously been reported by Reyes-Velasco et al. (2018). When only the 16s gene is analysed, the majority of the same groups were recovered; however, most of the nodes received poor support (bootstrap < 50; Suppl. material 3).

The uncorrected pairwise genetic distances between lineages in the mitogenome dataset ranged from 5.8% to 6.6% (Suppl. material 5: table S5), while those genetic distances, based on COX1, ranged from 5.3% to 10% (Suppl. material 5: table S6). These differences correspond to levels of inter-specific divergences previously reported in anurans (Smith et al. 2008).

### ﻿Phylogenetic placement of the type specimens of *Pseudocassinaocellata* and *P.rugosa* in the *Leptopelisgramineus* species complex

The phylogenetic analysis of the whole mitogenomes placed the types of *Pseudocassinaocellata* and *P.rugosa* as sister to one another and together sister to an individual from Mehal Meda (Semien Shewa Zone, Amhara Region; Suppl. material 1: fig. S1A; see phylogenetic analyses above). These relationships received strong support (bootstrap support = 100). These samples formed the sister group to all other individuals, which consisted of a specimen from the Harenna forest (*Leptopelisdiffidens*) and multiple individuals from the Bale Mountains and Assela (Suppl. material 1: fig. S1A). The relationships amongst individuals did not change when we only included the COX1 gene (Suppl. material 1: fig. S1B).

In both COX1 and 16s phylogenies, the type specimens of *Pseudocassinaocellata* and *P.rugosa* grouped with individuals from northwest of the GRV (“Northern” clade of Reyes-Velasco et al. 2018), with strong support (bootstrap support > 95; Fig. 2, Suppl. materials 2, 3). These results show that the types of *Pseudocassinaocellata* and *P.rugosa* are conspecific. Both names are, thus, synonyms and correspond to the semi-fossorial *Leptopelis* found predominantly in the plateaus and mountains west of the GRV.

### ﻿Linear morphometrics

Due to sexual dimorphism, we analysed male and female morphological measurements separately (Fig. 3; Table 1). The six species analysed morphologically can be split into three morphotype categories: a large, fully arboreal form, small, mostly arboreal forms and large, semi-fossorial forms. *Leptopelissusanae* is a truly arboreal form and can be distinguished from all other species of the group by enlarged finger and toe discs (FinDW and Toe4DW) and elongated hind-limbs (TL and THL; Suppl. material 5: table S9). Individuals from the Northern and Bale/Assela clades are semi-fossorial and distinguished from *L.gramineus*, *L.diffidens* and Kibre Mengist clade’s individuals by a greater body size (SVL), a greater tympanum diameter (TD), longer snout (SL), inter-nares (IND) and snout-nostril distance (SN) and metatarsal tubercle length (MTL; Suppl. material 5: table S9). The Northern clade can be distinguished from the Bale/Assela clade by longer hind-limbs (TL and THL) and larger finger and toe discs (FinDW and Toe4WD; Suppl. material 5: table S9).

Within the arboreal group, head shape is most discriminant between species. The Kibre Mengist clade can be distinguished from the other small species of the complex (*L.gramineus* and *L.diffidens*) by a longer snout-nostril (SN) and a shorter inter-orbital distance (IOD). *Leptopelisgramineus* has a greater inter-nares (IND) and shorter snout-nostril (SN) distance than *L.diffidens* and the Kibre Mengist clade (Suppl. material 5: table S9).

### ﻿Acoustic analysis

Advertisement calls of the *Leptopelisgramineus* species complex sound like a short rattle and species are difficult to distinguish by an untrained ear, except for *L.susanae*, which produces a call shorter in duration than the other species (Fig. 4; Table 2). The major differences between species lie in the number of pulses per note and pulse rate (Fig. 4; Table 2). The larger forms (*L.susanae*, the Northern and Bale/Assela clades) produce notes with fewer pulses than the smaller forms (*L.gramineus*, *L.diffidens* and the Kibre Mengist clade): 3–5 pulses vs. 5–9. These pulses are well spaced in the Northern clade (61 ± 14 pulses⋅s^–1^) while they are emitted in very quick succession by males from the Bale Mountains and Assela clade (88 ± 3 pulses⋅s^–1^) and *L.susanae* (209 ± 20 pulses⋅s^–1^). Kibre Mengist males produces notes with lower pulse rate (39 ± 7 pulses⋅s^–1^) than *L.gramineus* and *L.diffidens* (62 ± 9 and 53 ± 8 pulses⋅s^–1^, respectively). Finally, the notes of *L.diffidens* are longer (130 ± 21 ms) than those of *L.gramineus* (93 ± 6 ms).

### ﻿Systematic accounts

#### Validity of the name *Leptopelisrugosus* (Ahl, 1924)

In 1924, Ahl described *Pseudocassinarugosa* and *Pseudocassinaocellata* as distinct species in the same article. Both names were subsequently synonymised with *Leptopelisgramineus* by Largen in 1977. In 2021, Tiutenko and Zinenko resurrected *L.ocellatus* from synonymy with *L.gramineus*, but because a senior homonym, *Leptopelisocellatus* (Mocquard, 1902), existed, they assigned a new name to the species, *Leptopelismontanus*Tiutenko and Zinenko 2021. In the present study, we demonstrate that *Pseudocassinarugosa* Ahl, 1924 and *Pseudocassinaocellata* Ahl, 1924 (= *Leptopelismontanus*Tiutenko and Zinenko 2021) are synonyms. According to articles 60.1 and 60.2 of the International Code of Zoological Nomenclature, a junior homonym must be rejected and replaced by an available and valid synonym, if one exists. If multiple potentially valid synonyms exist, the oldest of these become the valid name. Therefore, being older than *Leptopelismontanus*Tiutenko and Zinenko 2021, *Leptopelisrugosus* (Ahl, 1924) becomes the valid name of the taxon.

##### ﻿ Leptopelis rugosus

Taxon classificationAnimaliaAnuraArthroleptidae

(Ahl, 1924)

399B4B5B-CC03-5643-9F52-0B7C70952E91

Pseudocassina rugosa Ahl, 1924.
Pseudocassina ocellata Ahl, 1924.
Leptopelis montanus Tiutenko & Zinenko, 2021.

###### Type material.

***Holotype*.** Adult female (ZMB–26915) collected during Oscar Neumann’s and Carlo von Erlanger’s expedition in 1900 in Meta, Kolla (approx. 9.17°N, 38.25°E, 2650 m a.s.l.).

###### Material examined.

In addition to the holotype, we examined one female collected at the end of July 1900 during O. Neumann’s and C. von Erlanger’s expedition in “Hochebene Didda” (ZMB-26913), which is one of the two syntypes of *Leptopelismontanus* Tiutenko & Zinenko, 2021 (= *Pseudocassinaocellata*), synonymised here with *L.rugosus*. As this specimen was mentioned as “the holotype” by Largen (2001) and has a collection date and a more precise locality than the other syntype (ZMB-26914), which only states “Somaliand”, considered erroneous by (Largen 1977), we formally designate ZMB-26913 as the lectotype of *Pseudocassinaocellata* here (see remarks below regarding the locality and taxonomic status). We also examined one female (16–130), collected on 12 July 2016 by J. Reyes-Velasco and S. Boissinot east of Mehal Meda (10.3316°N, 39.7812°E, 3265 m a.s.l.), one male (16–103), collected on 12 July 2016 by J. Reyes-Velasco and S. Boissinot north of Debre Sina (9.9894°N, 39.7452°E, 3017 m a.s.l.), three males (16–109, 16–116, 16–129), collected on 12 July 2016 by J. Reyes-Velasco and S. Boissinot east of Mehal Meda (10.16–10.33°N, 39.76–39.80°E, 3167–3265 m a.s.l.), five males (16–150, 16–151, 16–152, 16–153, 16–154), collected on 13 July 2016 by J. Reyes-Velasco and S. Boissinot east of Debre Birhan (9.6979°N, 39.5628°E, 2833 m a.s.l.), three males (16–164, 16–168, 16–172), collected on 14 July 2016 by J. Reyes-Velasco and S. Boissinot south of Fiche (9.73–9.75°N, 38.74°E, 2657–2726 m a.s.l.), five males (SB530, SB531, SB532, SB533, SB540), collected on 1 and 2 July 2018 by S. Goutte and Y. Bourgeois east of Debre Birhan (9.6979°N, 39.5628°E, 2339 m a.s.l.), four males (SB541, SB542, SB544, SB545), collected on 2 July 2018 by S. Goutte and Y. Bourgeois between Debre Birhan and Ankober (9.6820°N, 39.7390°E, 3408 m a.s.l.), two females (SB555, SB558) and one male (SB556), collected on 4 July 2018 by S. Goutte and Y. Bourgeois east of Mehal Meda (10.31–10.33°N, 39.78–39.80°E, 3337–3429 m a.s.l.) and three males (SB608, SB609, SB610), collected on 11 July 2018 by S. Goutte and Y. Bourgeois south of Fiche (9.7307°N, 38.7439°E, 2365 m a.s.l.).

###### Diagnosis.

**Figure 5.**
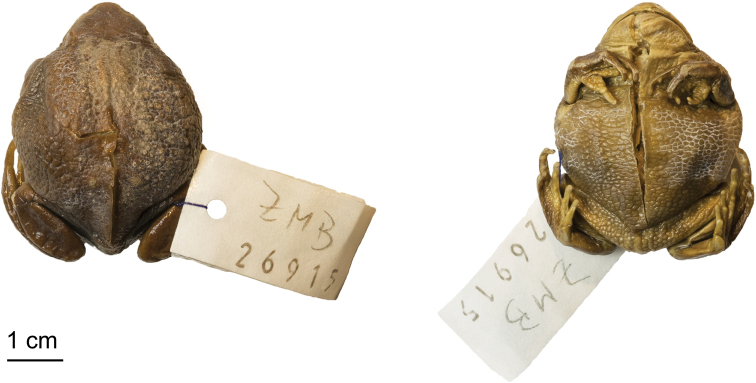
Dorsal and ventral views of the female holotype (ZMB-26915) of *Leptopelisrugosus*.

**Figure 6.**
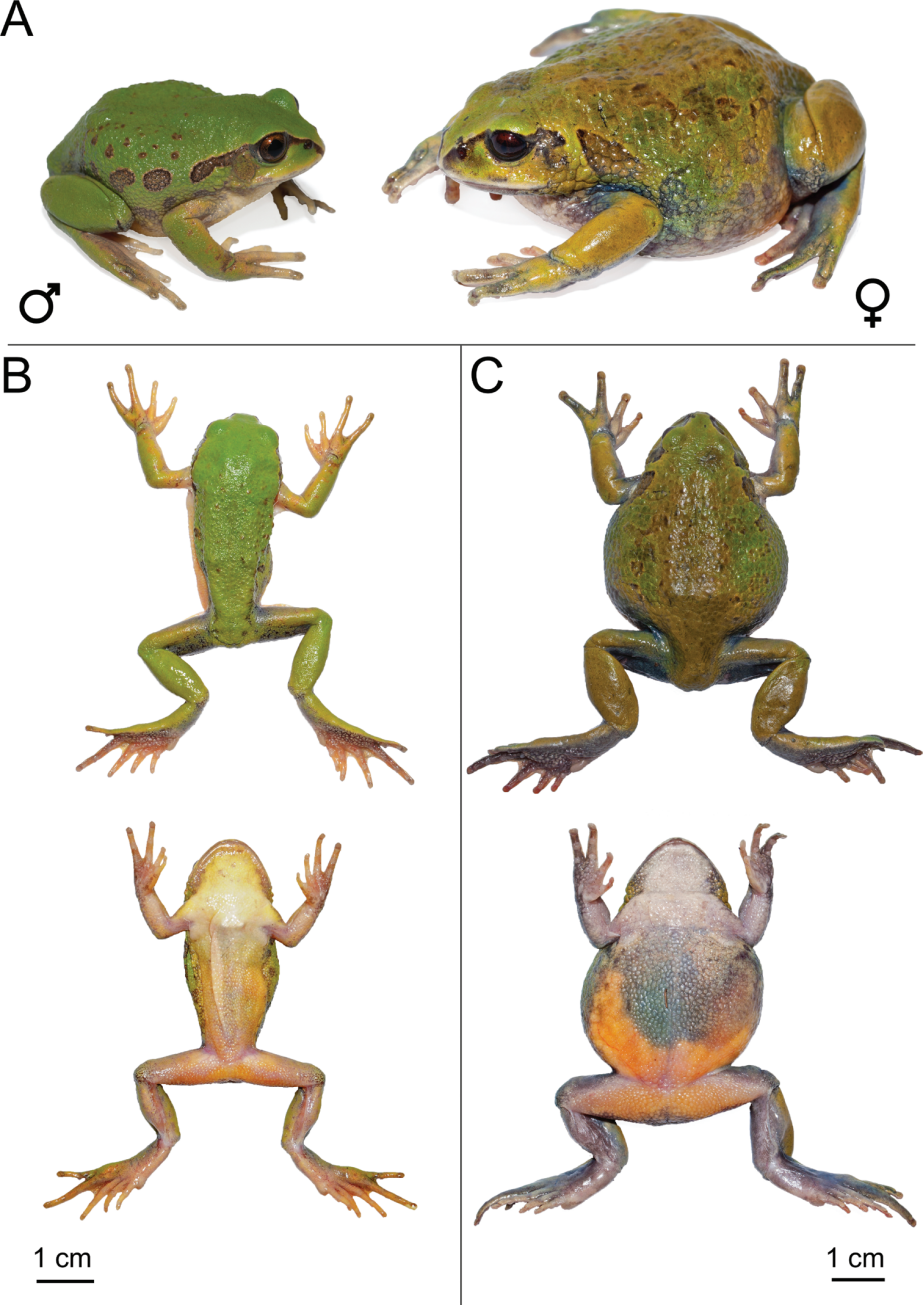
*Leptopelisrugosus***A** live male (SB610; left) and female (SB558; right) **B** dorsal and ventral views of male (SB610) after euthanasia and before fixation **C** dorsal and ventral views of female (SB558) after euthanasia and before fixation.

A large (male (n = 22) SVL 38.7 ± 2.7 mm, female (n = 2) SVL 52.4 ± 0.2 mm) species of the *Leptopelisgramineus* species complex (Figs 5, 6). Robust, semi-fossorial form. It differs from other members of the *Leptopelisgramineus* species complex by the following combination of characters: (1) large tympanum (male TD/ED 0.57 ± 0.11, female TD/ED 0.54), (2) long snout (male SL/HL 0.25 ± 0.02, female SL/HL 0.27 ± 0.01), (3) well-developed metatarsal tubercle (male MTL/FL 0.17 ± 0.02, female MTL/FL 0.18 ± 0.02), (4) ventrum lacking any brown spots, (5) yellow colouration on the side of the ventrum and the inner thighs almost always present.

###### Comparison.

**Figure 7.**
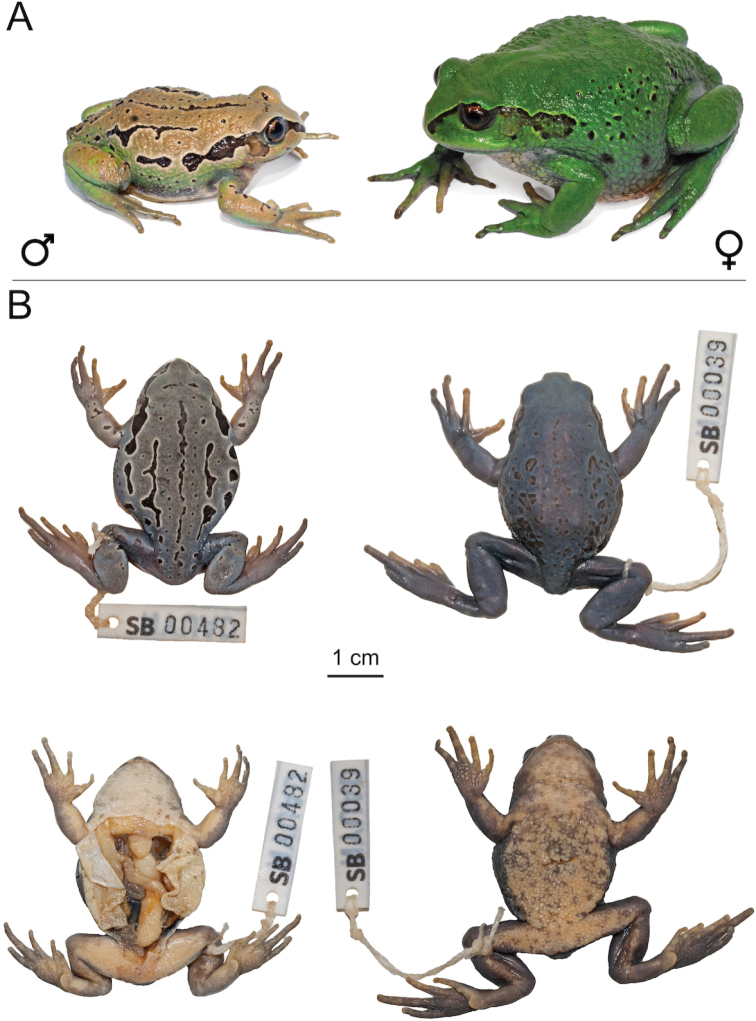
*Leptopelisshebellensis* sp. nov. **A** male holotype (SB482; left) and live female paratype (SB41; right) **B** male holotype (SB482; left) and dorsal and ventral views of female paratype (SB39; right) after fixation.

Larger body size, longer head and snout and greater snout-nostril distance and larger tympanum and metatarsal tubercle than *L.gramineus*, *L.diffidens* and *L.* sp. Kibre Mengist (Table 1, Suppl. material 5: table S9). Longer hind-limbs than the Bale/Assela clade, but shorter than *L.susanae* (Table 1, Suppl. material 5: table S9). Finger and toe discs less developed and head narrower and shorter than *L.susanae* (Table 1, Suppl. material 5: table S9). *Leptopelisrugosus* is distinguished from the Bale/Assela clade by the lack of dark pigmentation on the ventrum, throat and ventral side of the limbs (Figs 6, 7).

###### Description of the holotype.

Relatively large adult female (SVL 44.7 mm) in good condition of preservation (Fig. 5). Body robust and round. Head a third of body size in length, wider than long (HW/HL 1.21). Canthus rostralis obtuse and snout rounded and wide (IND/IOD 0.96). Nostril half-way between the tip of the snout and the eye (NS/SL 0.53). Tympanum partially hidden by flank skin rugosities and barely visible. Hind-limbs relatively long (TL/SVL 0.38 and THL/SVL 0.43). Finger and toe discs barely expanded, but distinct, ovoid. Finger formula: I < II < IV < III. Hand free of webbing. Foot longer than tibia (FL/TL 1.3). Inner metatarsal tubercle well-developed, oval in shape, 0.20× foot length. Outer metatarsal tubercle absent. Toe formula: I < II < V < III < IV. Toe webbing formula (toe internal/external sides, number of phalanges webbed): Ie(1), IIi/e(1–1), IIIi/e(1–2), IVi/e(2–2), Vi(2). Skin of the dorsum, flanks and ventrum highly rugose.

###### Colouration of the holotype in preservative.

Dorsal ground colour and canthal region dark olive brown with no visible pattern, except for a large light brown blotch covering about a third of the dorsum (Fig. 5). This discolouration probably appeared during the specimen preservation and after its original description, where Ahl described the dorsum as “solid dark olive-brown”. The thin light yellowish line noted by Ahl (1924) to extend to the upper arm is mostly faded away behind the tympanic region. Upper lip and flanks light yellowish-brown. Throat, ventrum, ventral side of the thighs and tibias light yellowish-brown. Front and hind-limbs olive brown without any marking.

###### Variations.

*Leptopelisrugosus* presents less colour polymorphism than the smaller members of the *L.gramineus* species complex. Dorsum is green to dark green and can be completely uniform or have a few to many irregular brown blotches (Fig. 6). In some individuals, these blotches form an irregular dorsal stripe extending from the top of the head to the lower back. A dark brown to black bar covers the canthal region and extends behind the eye, over the tympanum and sometimes behind the arm junction. This canthal stripe is overlined by a thin, more or less visible yellowish line. Flanks are the same colour as the dorsal ground colour and, in all individuals examined, except for the female SB558, have more or less well-defined brown ocelli. Limbs are the same colouration as the dorsum and rarely have irregular brown markings. Upper lip may be cream or a lighter shade of green than the dorsum without any markings. Iris sand colour to brown. Tympanum may be uniformly green or partially covered by a brown blotch joining the brown bar behind the eye. Throat and chest uniformly white to pale yellow. Ventrum generally white or cream with light to deep yellow zones on the sides extending to the ventral side of the thighs. In some individuals, the ventrum may be completely yellow. In gravid females, yellow eggs are visible through the thick ventral skin. In most individuals, the palms of the hands, ventral and inner sides of the limbs show very little to no dark pigmentation. Some individuals have a few irregular brown or black blotches on the inner tibia, forearm, hand and foot. Dorsal skin may be smooth, slightly or very rugose.

###### Habitat, distribution and natural history.

*Leptopelisrugosus* is found in grassy meadows of the Ethiopian Highlands north of the GRV at mid- to high elevations (2,339–3,337 m a.s.l.). This species occurs notably near Debre Birhan, Debre Sina, Fiche, Addis Ababa, Holeta and Ambo (Fig. 1, Suppl. material 5: table S1). The northernmost population was found near Mehal Meda (10.3171°N, 39.8024°E), while the southernmost individuals were found between Ambo and Wonchi (8.9007°N, 37.8928°E). One of the two types in Ahl’s original description of *Pseudocassinaocellata* was collected on the Arussi Plateau, which, if the locality is correct, is the only known specimen east of the GRV (see remark on the type locality below).

Males are heard calling at night and sometimes during the day for extended periods of time. Advertisement calls are emitted from the ground, either on the grass or from a cavity in the ground or under a rock, generally near a stream or a flooded area. Several males were found calling from the banks of a completely dried-out stream, although it is unknown to us whether the reproduction period extends to the dry season or whether males keep calling only during shorter dry periods.

###### Advertisement call.

The call of *Leptopelisrugosus* is a short rattle composed of a single note of 65 ± 21 ms in duration, containing 4 ± 1 pulses (Fig. 4B). In most individuals, the two first pulses are emitted at very short intervals, while the subsequent pulses are more spaced (average inter-pulse interval 20 ± 5 ms). Other individuals produce notes with regularly-spaced pulses. Amplitude is highest at the beginning of the note and decreases gradually. Within a call bout, calls are spaced by 13 ± 7 seconds, often with an acceleration of the call rate from a call every 10 seconds to one call per second. Call dominant frequency is 1,769 ± 60 Hz, with a bandwidth of 742 ± 82 Hz.

The call of *Leptopelisrugosus* is distinguishable from the calls of *L.gramineus*, *L.diffidens* and the Kibre Mengist and Bale/Assela clades by its lower number of pulses per note and narrower frequency band width. It is further distinguished from the call of *L.gramineus*, *L.diffidens* and *L.* sp. Kibre Mengist by its shorter note duration and from *L.* sp. Kibre Mengist by its lower peak frequency and higher pulse rate. Finally, it is distinguished from the call of *L.susanae* by its longer duration and lower pulse rate (Table 2).

###### Remarks.

Diagnostic characters used in the original description.

Ahl described *Pseudocassinarugosa*, based on a single female and provided three main diagnostic characters when compared with *Pseudocassinaocellata*, which he described in the same article, based on one female and one male: (1) the texture of the skin (rugose for *P.rugosa* and smooth for *P.ocellata*), (2) the length of the tibia (*P.rugosa*TL/SVL 1/3.5 and *P.ocellata*TL/SVL 1/3), (3) the visibility of the tympanum (hidden for *P.rugosa* and visible for *P.ocellata*). Ahl also named *P.ocellata* after the presence of ocelli on the flanks of the individuals he examined, which reflects on its specificity, even though he did not use this trait as a diagnostic character.

We found individuals of *L.rugosus* and the Bale/Assela clade with either a completely smooth, slightly rugose or coarsely rugose dorsum. The rugosity of the skin thus seems to be variable amongst individuals and, perhaps, age or season. Additionally, we have noticed that the rugosity of the skin may disappear after euthanasia and/or fixation of the specimen. Finally, even though almost all individuals of *L.diffidens*, *L.gramineus* and the *L.* sp. Kibre Mengist examined had smooth skin, we found two females *L.diffidens* and one female *L.* sp. Kibre Mengist with slightly rugose skin. The visibility of the tympanum seems to be variable across the individuals as well, perhaps linked to the size of the individual and the rugosity of the skin. While some female *L.rugosus* have a partially hidden tympanum, all males examined had a visible tympanum. The difference in tibia length between the specimens described by Ahl reflects individual variations as shown in our dataset. Finally, ocelli on the flanks and/or dorsum are present in certain individuals of *L.rugosus* and the Bale/Assela clade and is not a diagnostic character.

#### Type localities of *Pseudocassinaocellata* and *P.rugosa*

Ahl described *Pseudocassinaocellata*, based on one female and one male, collected at different localities during Oscar Neumann’s and Carlo von Erlanger’s 1900 expedition. The information given for the female is “Hochebene, Didda, end of July 1900”, while for the male, only “Somaliland” is given. Although imprecise, both localities are clearly situated east of the Great Rift Valley (GRV). In contrast, the specimen used as holotype for the original description of *P.rugosa* and all recent *L.rugosus* specimens were collected west of the GRV. We propose two, non-exclusive explanations for this discrepancy.

First, based on Neumann’s report and map of the expedition (Neumann 1902) and the collection date, “End of July 1900”, the locality of collection of the first type of *P.ocellata* could be near Addis Ababa and not on the Arussi Plateau (i.e. Didda Plateau). Indeed, the expedition party crossed the Shebelle River the first time near Jabolo, east of Sheik Hussein on 10 June, continued west through Sheik Hussein, ascended a couple of mountains, crossed the Shebelle River a second time and ascended the Arussi Plateau. Neumann reports that it took them 12 days to cross the Arussi Plateau, after which they descended into the flooded Awash Valley and reached Addis Ababa from the southeast on 14 August 1900. Depending on their pace between Jabolo and the eastern edge of the Arussi Plateau and when ascending to Addis Ababa, at the end of July 1900, the expedition party could have been anywhere between the western Arussi Plateau and Addis Ababa. Notably, the specimen could have been collected on the Yerer Mountain, southeast of the capital and culminating above 2,800 m a.s.l.

Second, although the distribution range of *L.rugosus* is mostly west of the GRV, it is possible that a population occupies the west Arussi Plateau (or did in 1900). We found individuals of the grass frog *Ptychadenabeka* Goutte, Reyes-Velasco, Freilich, Kassie and Boissinot 2021 at the north-western edge of the Arussi Plateau, even though the distribution range of this species is otherwise exclusively west of the GRV. We also found in the same area individuals of the river frog *Amietianutti* (Boulenger 1896) carrying a mitochondrial haplotype restricted to the west of the GRV (Manthey et al. 2017). It is, thus, possible that conditions recently permitted the crossing of the GRV in that area for several anuran species and that *L.rugosus* is present or was recently present, east of the GRV.

##### ﻿ Leptopelis shebellensis

Taxon classificationAnimaliaAnuraArthroleptidae

Goutte, Reyes-Velasco, Kassie & Boissinot sp. nov.

07AF3730-BB60-5C08-93F9-A911DBAF0383

https://zoobank.org/C3219B8F-7580-476A-BA0D-44F8D0822400

###### Common name.

English: Shebelle River burrowing African treefrog.

###### Type material.

***Holotype*.** Adult male (SB482), collected on 26 June 2018 by S. Goutte and Y. Bourgeois near the town of Ch’ange, Oromia, Ethiopia (8.1263°N, 39.4360°E, 2429 m a.s.l.). ***Paratypes*.** One male (15–46), collected on 5 August 2015 by X. Freilich, J. Reyes-Velasco and S. Boissinot, south of Assela (7.9068°N, 39.1238°E, 2520 m a.s.l.), one male (15–79) and one female (15–83), collected on 6 August 2015 by X. Freilich, J. Reyes-Velasco and S. Boissinot, southwest of Dinsho (7.1156°N, 39.7390°E, 3029 m a.s.l.), one male (15–84), collected on 6 August 2015 by X. Freilich, J. Reyes-Velasco and S. Boissinot southwest of Dinsho (7.1105°N, 39.7461°E, 3042 m a.s.l.), one male (15–143), collected on 8 August 2015 by X. Freilich, J. Reyes-Velasco and S. Boissinot between Robe and Ali (7.1720°N, 39.9722°E, 2431 m a.s.l.), one female (15–152), collected on 8 August 2015 by X. Freilich, J. Reyes-Velasco and S. Boissinot in Goba (7.0110°N, 39.9677°E, 2699 m a.s.l.), two females (16–8, 16–9), collected on 5 July 2016 by J. Reyes-Velasco and S. Boissinot south of Assela (7.8656°N, 39.1305°E, 2605 m a.s.l.), one females (16–25), collected on 6 July 2016 by J. Reyes-Velasco and S. Boissinot south of Assela (7.8836°N, 39.1245°E, 2531 m a.s.l.), two males (16–26, 16–28), collected on 6 July 2016 by J. Reyes-Velasco and S. Boissinot north of Bekoji (7.5585°N, 39.2520°E, 2721 m a.s.l.), one male (16–88), collected on 10 July 2016 by J. Reyes-Velasco and S. Boissinot east of Dinsho (7.1065°N, 39.8184°E, 3065 m a.s.l.), one male (16–93), collected on 10 July 2016 by J. Reyes-Velasco and S. Boissinot west of Dinsho (7.1204°N, 39.7358°E, 3048 m a.s.l.), three males (SB61, SB62, SB63), collected on 26 June 2018 by S. Goutte and J. Reyes-Velasco south of Dinsho (7.0915°N, 39.7834°E, 3079 m a.s.l.), two males (SB483, SB484), collected on 26 June 2018 by S. Goutte and Y. Bourgeois near Ch’ange (8.1263°N, 39.4360°E, 2429 m a.s.l.), three males (SB502, SB504, SB505), collected on 28 June 2018 by S. Goutte and Y. Bourgeois north of Arussi Robe (7.9190°N, 39.6091°E, 2433 m a.s.l.).

###### Diagnosis.

Medium to large (male (n = 21) SVL 35.9 ± 3.5 mm, female (n = 5) SVL 53.4 ± 5.3 mm), robust semi-fossorial species of the *Leptopelisgramineus* species complex (Fig. 7). It differs from other members of the *Leptopelisgramineus* species complex by the following combination of characters: (1) short and robust hind-limbs (male TL/SVL 0.33 ± 0.03, female TL/SVL 0.30 ± 0.02), (2) well-developed metatarsal tubercle (male MTL/FL 0.17 ± 0.03, female MTL/FL 0.16 ± 0.02), (3) longer snout (male SL/HL 0.23 ± 0.03, female SL/HL 0.24 ± 0.02), (4) toe and fingertips not enlarged and (5) ventrum often with dark brown spots and/or yellowish sides.

###### Comparison.

Larger body size, longer head and snout and greater snout-nostril distance and larger tympanum and metatarsal tubercle than *L.gramineus*, *L.diffidens* and *L.* sp. Kibre Mengist (Table 1, Suppl. material 5: table S9). Shorter hind-limbs and smaller finger and toe discs than *L.rugosus* and *L.susanae* (Table 1, Suppl. material 5: table S9).

###### Description of the holotype.

Medium size adult male (SVL 40.6 mm). Body robust (Fig. 7). Head a third of body size in length, wider than long (HW/HL 1.14). Snout wide (IND/IOD 0.87). Nostrils closer to the tip of the snout than the eyes (NS/SL 0.47). Canthus rostralis well-marked, but obtuse and loreal region slightly concave. Pupil vertical. Tympanum partially hidden on the posterior-dorsal edge, 0.40× eye diameter.

Fingers and toes robust with ovoid discs not expanded, but distinct. Finger formula: I <II < IV < III. Hand free of webbing. Hind-limbs short and robust (TL/SVL 0.31 and THL/SVL 0.38). Foot 1.32× tibia length. Inner metatarsal tubercle present, oval in shape, 0.16× foot length. Outer metatarsal tubercle absent. Toe formula: I < II < III < V < IV. Foot webbing minimal, except between toe III and toe IV where webbing extends to half-way between the first and the second phalanges. Skin of the dorsum, flanks and ventrum slightly rugose.

###### Colouration of the holotype in life.

Dorsal ground colour sand, slightly iridescent, with green hues in the lower two-thirds (Fig. 7). One dorsal and two latero-dorsal irregular dark brown bands outlined with a thin cream line. Several small round or ovoid dark-brown spots, outlined with a thin cream line, are present in between the dorsal and latero-dorsal stripes and two larger and irregular in shape are present between the eyes. Dark brown canthal stripe, outline with a cream-coloured line, from the tip of the snout extending above and around the tympanum and behind the shoulder on the right side and to a fourth of the abdomen on the left side. Several large and irregular dark brown blotches, outlined by a cream line on the flank, in the continuation of the canthal stripe on each side. Tympanum golden light brown. Upper lip iridescent sand colour with a few irregular brown markings. Iris dark gold, lighter on the upper third, with heavy black reticulation. Flanks sand colour dorsally to forest green ventrally, with small black round spots. An irregular dark grey blotch marks the limit between the flank and the ventrum. Throat and ventrum mostly cream, yellowish towards the flanks, with small light brown blotches laterally. Hands, arms and forearms sand colour to green posteriorly, with a few irregular dark brown spots, except on the hands. Tibia light brown with irregular green blotches and a few small and irregular black spots. Thighs green dorsally to dark, bluish-green posteriorly, with a few irregular brown blotches. Feet green towards the heel to light olive green towards the toes.

###### Colouration of the holotype in preservative.

Dorsal ground colour bluish-grey with large irregular black bands and spots outlined by a white line (Fig. 7B). Hands, feet and limbs bluish-grey with a few irregular black spots on the forearms and tibias, outlined with white. Throat and ventrum white to cream with a few faint brown spots. Ventral side of the hands, feet and tibiae heavily dusted with grey.

###### Variation.

Dorsum can be green to dark green, sand or brown. All examined specimens had light or dark brown to black irregular markings, variable in size and number, on the dorsum. In many individuals, those markings are bi- or tricolour (yellowish-cream, light and dark brown) and form lateral and dorso-lateral ocelli in some animals. A thin yellowish line is present from the tip of the snout to behind the tympanum in all examined individuals. The canthal stripe can be light brown to black and can be underlined by a second yellowish line from the snout to the eye in some individuals. Flanks can be the same colour or a lighter version of the dorsal ground colour or green while the dorsum is brown or vice versa. Larger versions of the dorsal blotches are found on the flanks, sometime merging into an irregular band. Limbs are the same colouration as the dorsum and sometimes have irregular brown markings. Upper lip may be light brown or the same colour as the dorsum without any markings. Iris golden to brown. Tympanum partially or completely covered with a brown blotch, either joining the brown bar behind the eye or as a separate blotch. Throat and chest uniformly white to pale yellow. Ventrum white to orange-yellow, sometimes with yellow to orange zones on the sides and extending to the ventral side of the thighs. In gravid females, yellow eggs are visible through the thick ventral skin. Palms of the hands, ventral sides of feet and tibia more or less heavily dusted with dark grey. Dorsal skin may be smooth, slightly or very rugose.

###### Etymology.

The specific name refers to the Shebelle River, as the distribution range of the species appears restricted to the Shebelle River Basin, with populations found both north and south of the source of the river (Fig. 1).

###### Habitat, distribution and natural history.

*Leptopelisshebellensis* sp. nov. inhabits the grassy meadows of the Didda Plateau and the northern Bale Mountains at mid- to high elevations (2,429–3,296 m a.s.l.). This species is notably found near Assela, Huruta, Dinsho, Adaba, Dodola, Goba and Chole (Fig. 1; Suppl. material 5: table S1). Males have been heard calling both during the dry (April, June) and rainy seasons (July, August) and call both at night and during the day, for extended periods of time. Males call on the bank of streams or side pools, generally from holes in the ground, sometimes from the ground under low vegetation.

###### Advertisement call.

The call of *Leptopelisshebellensis* sp. nov. is a very short rattle. It is composed of one or two identical notes at 704 ± 85 ms interval (Fig. 4A). When they are produced, two-note calls make up about half of the calls within a call bout. Each note is 57 ± 5 ms in duration and contains five pulses, emitted regularly with very short inter-pulse intervals (11 ± 2 ms). The relative position of the note’s amplitude peak is variable amongst individuals and may be on the first pulse, in the middle of the note or the amplitude may be equivalent for each pulse. Within a call bout, calls are spaced by 8 ± 1 seconds. Call dominant frequency is 1,616 ± 265 Hz, with a band width of 974 ± 163 Hz.

The call of *Leptopelisshebellensis* sp. nov. is distinguishable from the calls of *L.gramineus*, *L.diffidens*, *L.* sp. Kibre Mengist and *L.rugosus* by its higher pulse rate. It is further distinguished from the call of *L.gramineus*, *L.diffidens* and *L.* sp. Kibre Mengist by its shorter note duration and narrower frequency band width and from *L.susanae* by its longer note duration and lower pulse rate. Finally, it can be distinguished from the call of *L.rugosus* by its greater number of pulses per note.

##### ﻿ Leptopelis xeniae

Taxon classificationAnimaliaAnuraArthroleptidae

Goutte, Reyes-Velasco, Kassie & Boissinot sp. nov.

45DB2E04-856C-5F1B-9153-298EE20770DE

https://zoobank.org/1773A2C3-8562-4F29-96E3-7C809FD7FD74

###### Common name.

English: Xenia’s African treefrog.

###### Type material.

***Holotype*.** Adult male (SB183), collected on 18 April 2018 by S. Goutte and J. Reyes-Velasco east of Kibre Mengist (5.8782°N, 39.1330°E, 1832 m a.s.l.). ***Paratypes*.** One female (SB151) and two males (SB152, SB153), collected on 17 April 2018 by S. Goutte and J. Reyes-Velasco northwest of Kibre Mengist (5.9988°N, 38.8798°E, 2097 m a.s.l.), four males (SB167, SB168, SB169, SB170), collected on 17 April 2018 by S. Goutte and J. Reyes-Velasco northwest of Kibre Mengist (6.0093°N, 38.8576°E, 2105 m a.s.l.) and one male (SB184) collected on 18 April 2018 by S. Goutte and J. Reyes-Velasco east of Kibre Mengist (5.8782°N, 39.1330°E, 1832 m a.s.l.).

###### Other material examined.

One female (SB197) and 12 males (SB186–SB196, SB206), collected on 19 April 2018 by S. Goutte and J. Reyes-Velasco southeast of Kofele (7.0226°N, 38.8701°E, 2,561 m a.s.l.).

###### Diagnosis.

**Figure 8.**
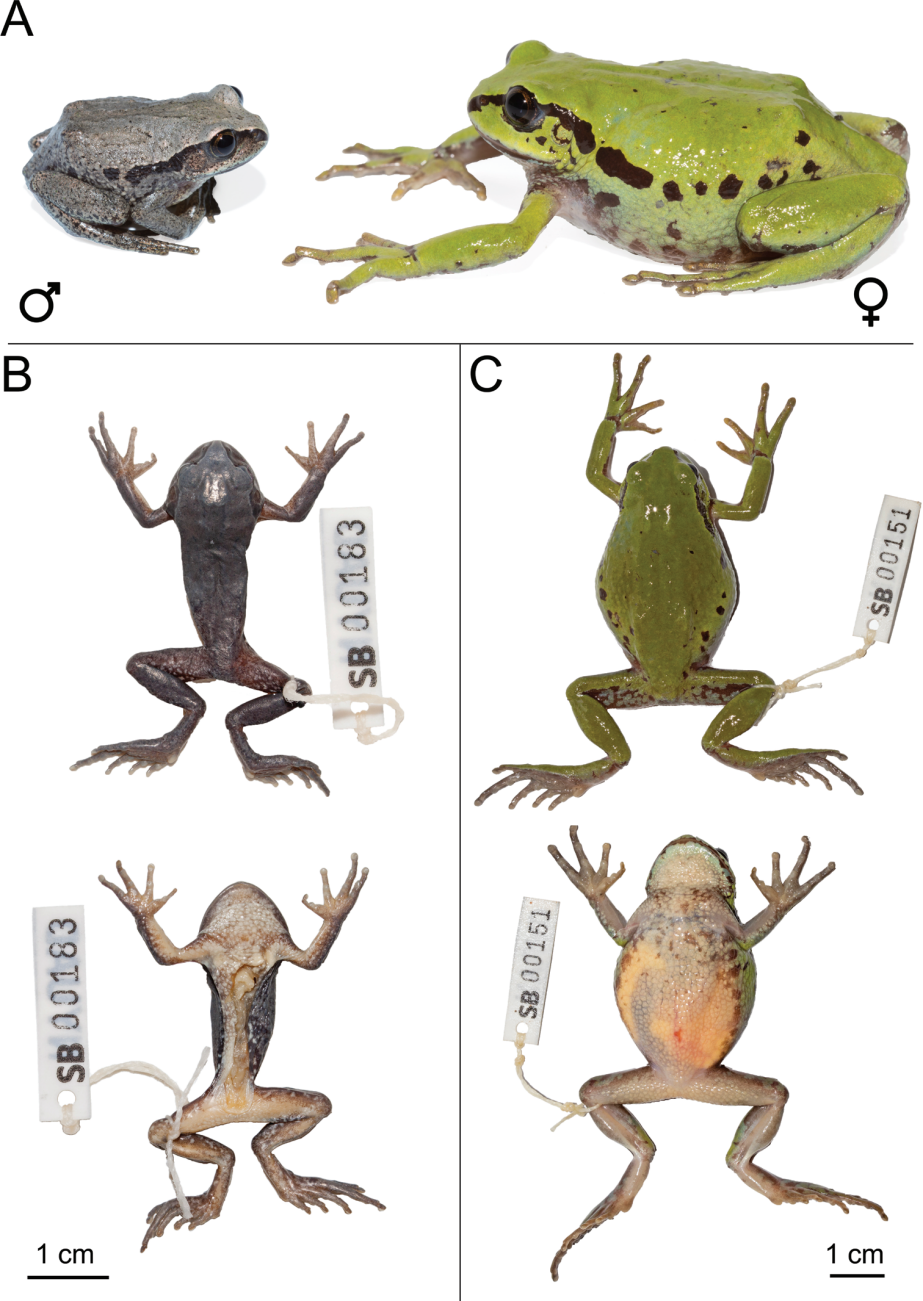
*Leptopelisxeniae* sp. nov. **A** live male holotype (SB183; left) and female paratype (SB151; right) **B** dorsal and ventral views of the male holotype (SB183) after fixation **C** dorsal and ventral views female paratype (SB151) after euthanasia and before fixation.

Small to medium-sized (male (n = 20) SVL 27.6 ± 2.0 mm, female (n = 2) SVL 43.5 ± 5.7 mm), robust arboreal species of the *Leptopelisgramineus* species complex (Fig. 8). It differs from other members of the *Leptopelisgramineus* species complex by the following combination of characters: (1) thin, elongated hind-limbs (male TL/SVL 0.36 ± 0.02, female TL/SVL 0.37 ± 0.03), (2) small metatarsal tubercle (male MTL/SVL 0.07 ± 0.01, female MTL/SVL 0.06 ± 0.00) (2) inter-orbital distance very short (male IOD/ED 0.88 ± 0.16, female IOD/ED 0.69 ± 0.12), (3) dorsal skin always smooth, except in females where it may be slightly rugose, (4) absence of yellow colouration on the ventrum or inner thighs.

###### Comparison.

Smaller body size, narrower head than *L.rugosus*, *L.shebellensis* sp. nov. and *L.susanae* (Table 1, Suppl. material 5: table S9). Tympanum and metatarsal tubercle smaller than *L.rugosus* and *L.shebellensis* sp. nov. Interorbital distance shorter than *L.gramineus* and *L.diffidens*. Snout narrower than *L.gramineus*, *L.rugosus* and *L.shebellensis* sp. nov. and more elongate (snout-nostril distance greater) than *L.gramineus* and *L.diffidens*. Hind-limbs and feet longer than in *L.gramineus*, *L.rugosus* and *L.shebellensis* sp. nov. (Table 1, Suppl. material 5: table S9).

###### Description of the holotype.

Small-sized male (SVL 28.9 mm) adult (Fig. 8). Body robust. Head a third of body size in length, wider than long (HW/HL 1.16). Snout angular and narrow (IND/IOD 0.75). Nostril beyond half-way between the tip of the snout and the eye (NS/SL 0.56). Canthus rostralis well-marked and loreal region slightly concave. Pupil vertical. Tympanum clearly visible, and round, 0.71× eye diameter. Fingers and toes thin and elongated with discs barely expanded, but distinct, ovoid. Finger formula: I < II < IV < III. Hand free of webbing. Hind-limbs relatively long for the *L.gramineus* species complex (TL/SVL 0.38 and THL/SVL 0.42 vs. TL/SVL < 0.37 and THL/SVL < 0.40 in other species of the *L.gramineus* complex, except *L.susanae*). Foot 1.2× tibia length. Inner metatarsal tubercle present, oval in shape, 0.21× foot length. Outer metatarsal tubercle absent. Toe formula: I < II < V < III < IV. Foot webbing minimal. Skin of the dorsum and flanks smooth, ventrum rugose.

###### Colouration of the holotype in life.

Dorsal ground colour grey-brown with three dark, but very faint, wide bands; the central band forming a triangle pointing towards the snout and each eye and extending along the spine to about three-fourths of the dorsum (Fig. 8A). Two similarly-coloured symmetrical bands on each side, starting behind the shoulder and fading away at the same level as the dorsal stripe. A few small dark brown spots on the head and dorsum. Black canthal stripe from the tip of the snout extending above the tympanum and behind the shoulder on the left side and to a third of the abdomen on the right side. One large black blotch on the flank, in the continuation of the canthal stripe on each side. Tympanum light grey dusted with small black spots. Upper and lower lip light grey with small irregular black markings. Iris dark gold, lighter on the upper third, with heavy black reticulation. Flanks grey, yellow towards the thighs, with irregular black blotches. Ventrum and throat cream, reticulated with light grey on the chest. Limbs, hands and feet grey dusted with small black freckles. Back of the thighs dark brown with irregular bluish-grey and yellowish-grey markings.

###### Colouration of the holotype in preservative.

Dorsal ground colour grey with a few small irregular dark grey spots (Fig. 8B). The faint three-bands pattern formed by these small dark grey spots is almost completely undistinguishable. Hands, feet and limbs grey with a few irregular dark grey spots. Back of the thighs dark brown with irregular light grey blotches. Throat and ventrum cream with some light brown or grey dusting. Ventral side of the thighs cream. Ventral side of the hands, feet and tibiae heavily dusted with brown and with a few irregular white spots.

###### Variations.

As with other members of the *Leptopelisgramineus* species complex, *L.xeniae* sp. nov. shows significant colour polymorphism. Dorsal colouration varies from light grey (with or without some light green) or light brown to bright or dark green with important level of dark pigmentation. Most individuals examined display a similar dorsal pattern composed of three bands, which can be barely distinguishable to well-marked. The canthal stripe may extend as a wide dark brown to black stripe on the flank to four-fifths of the abdomen or be replaced by large blotches of the same colour. Limbs are the same colouration as the dorsum, with variable number of small to medium brown or black markings. Upper lip may be cream, light grey or green, with variable size and number of irregular brown or black markings. Iris golden to dark golden. Tympanum dark brown or black either entirely or on its upper half, with the lower half being the same colour as the dorsum. Rarely, the tympanum is entirely the same colour as the dorsum. Lower lip cream mottled with brown or grey or with a continuous brown blotch. In one female (SB151), the lower lip was light green with a few brown markings. Ventrum and throat white, with small brown blotches on either or both in most individuals. Inner thighs white or lacking any pigmentation, except dark brown and yellow spots towards the knee in some individuals. Cream-coloured eggs visible through the thick ventral skin of the gravid female SB151. Dorsal skin smooth in all specimens examined, except in the female SB197, which had a slightly rugose dorsal skin.

###### Etymology.

*Leptopelisxeniae* sp. nov. is named after Xenia Freilich, who conducted her doctoral research on Ethiopian anurans, including the *Leptopelisgramineus* complex.

###### Habitat, distribution and natural history.

*Leptopelisxeniae* sp. nov. is found in the forested areas around the towns of Kibre Mengist and Kofele, Oromia, Ethiopia (5.87–7.02°N, 38.80–39.13°E; Fig. 1, Suppl. material 5: table S1). The species occupies lower elevations than most other members of the *L.gramineus* species complex (1,832–2,561 m a.s.l.). Males are found calling at night from the ground or from vegetation up to 60 cm high, close to slow-flowing streams or in flooded forest clearings. Reproductive biology is unknown beyond the calling behaviour and we have not encountered eggs or tadpoles.

###### Advertisement call.

As for other members of the *Leptopelisgramineus* species complex, the call of *Leptopelisxeniae* sp. nov. is a short rattle (Fig. 4D). It is composed of a single note of 175 ± 25 ms in duration, containing 7 ± 2 pulses, which are clearly distinct. Within a note, pulses are spaced by intervals of 30 ± 5 ms. These inter-pulse intervals may be of equal length throughout the note or shorter between the last pulses. Amplitude is highest for the first quarter to half of the note and decreases in its last quarter. Within a call bout, calls are spaced by 14 ± 7 seconds, often with an acceleration of call rate starting with one call every 22 seconds to one note every four seconds. Call dominant frequency is 2,231 ± 585 Hz, with an important band width (2,013 ± 573 Hz).

The call of *Leptopelisxeniae* sp. nov. is distinguishable from the calls of *L.gramineus*, *L.diffidens*, *L.shebellensis* sp. nov., *L.rugosus* and *L.susanae* by its longer note and longer inter-pulse intervals (Fig. 4, Table 2). It is further distinguished from the call of *L.shebellensis* sp. nov. and *L.rugosus* by a greater number of pulses per note, a higher peak frequency and a wider frequency band width (Table 2).

#### Remark on the Kofele population

Around the town of Kofele (7.0226°N, 38.8701°E, 2,561 m a.s.l.; Fig. 1), we found a population of *Leptopelisxeniae* sp. nov., which carried the mitochondrial genome of *L.shebellensis* sp. nov. suggesting gene flow between the two species in this area (Fig. 2; Reyes-Velasco et al. 2018). Individuals from this population are morphologically indistinguishable from other *L.xeniae* sp. nov. individuals and clearly different from *L.shebellensis* sp. nov. (Fig. 3). In addition, their nuclear genome is mostly of *L.xeniae* sp. nov. origin (Reyes-Velasco et al. 2018). Since the Kofele population shows evidence of past hybridisation, individuals from this locality have been excluded from the type series.

#### Remarks and notes on *Leptopelisdiffidens*

*Leptopelisdiffidens* shows substantial colour polymorphism, with dorsal colouration ranging from light yellow, brown, sand, orange to bright or bluish-green. Many individuals have a more or less marked three-banded pattern also shared with other species of the genus. Contrary to what is stated in the original description, we found *L.diffidens* in syntopy with *Leptopelisragazzii* in multiple forest clearings, with males calling simultaneously. Male *L.diffidens* were found calling on flooded grass or reeds, while males *L.ragazzii* called from trees at 1.5–3 m above the ground. We also found multiple *L.diffidens* males calling on vegetation and under rocks around a rivulet running through the small town of Rira. Several female *L.diffidens* were found on shrubs as high as 1.5 m above the ground.

Beside the advertisement call produced by males, we recorded three calls, which we identified as aggressive calls, from two different males (Suppl. material 4: fig. S4A). These calls are longer than the species’ advertisement call (1050 ± 148 ms) and composed of three pulse groups of 4 ± 1, 4 ± 1 and 23 ± 1 pulses, respectively. The peak frequency of this call is lower than the advertisement call, at 1705 ± 34 Hz.

### ﻿Identification key

**Table:** 

1	Medium to large body size (male SVL 26–38 mm, female SVL 44–57 mm), finger and toe discs very enlarged, elongated hind-limbs (male TL/SVL 0.40–0.49, female TL/SVL 0.41–0.45; male THL/SVL 0.40–0.49, female THL/SVL 0.42–0.49	***L.susanae***
–	Small to large body size (male SVL 21.5–45 mm, female SVL 35.9–61 mm), finger and toe discs barely expanded or not expanded at all, hind-limbs short to moderately elongated	**2**
2	Medium to large body size (male SVL 30–45 mm, female SVL 45–61 mm), large tympanum (male TD/ED > 0.51, female TD/ED > 0.54), metatarsal tubercle spade-shaped and well developed	**3**
–	Small to medium body size (male SVL 21.5–38.8 mm, female SVL 35.9–50.2 mm), small tympanum (male TD/ED < 0.54, female TD/ED < 0.45), small ovoid metatarsal tubercle	**4**
3	Ventral area and inner limbs with very little to no melanisation	***L.rugosus***
–	Palms of the hands, ventral sides of feet and tibia more or less heavily dusted with dark grey	***L.shebellensis***
4	Inter-orbital distance very short (male IOD/ED 0.88 ± 0.16, female IOD/ED 0.69 ± 0.12), snout narrow and elongate (male SN/SL 0.56 ± 0.05, female SN/SL 0.57 ± 0.00	***L.xeniae***
–	Inter-orbital distance not very short (male IOD/ED > 0.76, female IOD/ED > 0.77) snout less narrow (male and female SN/SL < 0.53	**5**
5	Finger tips barely enlarged	***L.gramineus***
–	Finger tips enlarged	***L.diffidens***

## ﻿Discussion

In the present work, we resolved issues related to the taxonomy of the *Leptopelisgramineus* species complex, which consists of at least six species. We described two new species found east of the GRV and clarified the status of montane populations west of the GRV. Using genetic and morphometric analyses of type specimens, we demonstrated that *Leptopelisrugosus* (= *Pseudocassinarugosa)* and *L.montanus* (= *Pseudocassinaocellata*) are, in fact, conspecific with the population northwest of the GRV and that the mountain populations east of the GRV belong to a new species, *L.shebellensis* sp. nov. In this species complex, including historical type specimens was necessary to assign the correct names to newly-discovered and previously-synonymised taxa. The present case is reminiscent of the situation reported in Ethiopian *Ptychadena*, where the analysis of type specimens allowed us to clarify the convoluted taxonomy of the group (Goutte et al. 2021; Reyes-Velasco et al. 2021).

The *Leptopelisgramineus* species complex comprises species living in most of the biotopes present in the Ethiopian Highlands, from mid-elevation forests (~ 1,800 m a.s.l.) to high-elevation montane grasslands (> 3,200 m a.s.l.). Members of this clade show traits associated either with a semi-fossorial lifestyle (e.g. well-developed metatarsal tubercles, robust limbs and body shape) in species living in high elevation grasslands (*L.rugosus* and *L.shebellensis* sp. nov.) or with a more arboreal lifestyle (e.g. elongated limbs, slender body) in species inhabiting forests and forest edges (*L.diffidens*, *L.susanae* and *L.xeniae* sp. nov.). The *L.gramineus* species complex thus adds to a growing list of groups that have diversified in the Ethiopian Highlands (e.g. Goutte et al. 2019, 2021; Koppetsch 2020; Kostin et al. 2020; Soultan et al. 2020) and highlights the ecological importance of this unique region.

The restriction of *Leptopelisgramineus* to the south-eastern population near Chencha and description of several species previously thought to be *L.gramineus* should impact the conservation status of Ethiopian *Leptopelis*. Currently, *Leptopelissusanae* is listed as “Endangered” by the IUCN because of its limited distribution range, while *L.gramineus*, considered widespread in Ethiopia, is listed as “Least concern” (IUCN 2022). Other species of the *L.gramineus* species complex are not currently listed. Based on our findings and on ongoing deforestation and land-transformation for pasture and agriculture in the region (Dessie and Christiansson 2008; Assefa and Bork 2014; Kindu et al. 2015), we expect that several species of the *L.gramineus* complex will be listed as “Near threatened”, “Endangered” or “Vulnerable” under IUCN Criteria B1ab (IUCN Standards and Petitions Committee 2022). Thus, we hope that our clarification of the *Leptopelisgramineus* species complex’s taxonomy will encourage conservation efforts in the Ethiopian Highlands, particularly in currently unprotected areas overlapping with *Leptopelis* spp. ranges.

## Supplementary Material

XML Treatment for Leptopelis rugosus

XML Treatment for Leptopelis shebellensis

XML Treatment for Leptopelis xeniae

## Supplementary materials

Supplementary material 1 Maximum Likelihood phylogenetic inference Data type jpg file Explanation note **Figure S1. A** Maximum Likelihood phylogenetic inference, based on 13 mitochondrial protein coding genes and the two ribosomal RNAs (12s and 16s) for members of the *Leptopelisgramineus* complex. Names in bold represent historical type specimens of *Pseudocassinarugosa* (ZMB-26915) and *Pseudocassinaocellata* (ZMB-26913). Bootstrap values are indicated at the nodes. Circles represent nodes with bootstrap values of 100. **B** Maximum Likelihood phylogenetic inference, based on the protein coding gene COX1 for members of the *Leptopelisgramineus* complex with complete mtDNA genomes. Names in bold represent historical type specimens of *Pseudocassinarugosa* (ZMB-26915) and *Pseudocassinaocellata* (ZMB-26913). Bootstrap values are indicated at the nodes. Circles represent nodes with bootstrap values of 100. https://binary.pensoft.net/file/766802This dataset is made available under the Open Database License (http://opendatacommons.org/licenses/odbl/1.0/). The Open Database License (ODbL) is a license agreement intended to allow users to freely share, modify, and use this Dataset while maintaining this same freedom for others, provided that the original source and author(s) are credited.Sandra Goutte, Jacobo Reyes-Velasco, Abeje Kassie, Stéphane Boissinot

Supplementary material 2 Bayesian phylogenetic inference of the Leptopelisgramineus species complex, based on COX1 Data type jpg file Explanation note Posterior probabilities are given at the nodes. Names in bold represent historical type specimens of *Pseudocassinarugosa* (ZMB-26915) and *Pseudocassinaocellata* (ZMB-26913). https://binary.pensoft.net/file/766803This dataset is made available under the Open Database License (http://opendatacommons.org/licenses/odbl/1.0/). The Open Database License (ODbL) is a license agreement intended to allow users to freely share, modify, and use this Dataset while maintaining this same freedom for others, provided that the original source and author(s) are credited.Sandra Goutte, Jacobo Reyes-Velasco, Abeje Kassie, Stéphane Boissinot

Supplementary material 3 Maximum Likelihood phylogenetic inference, based on the ribosomal RNAs 16s for members of the *Leptopelisgramineus* complex Data type jpg file Explanation note Names in bold represent historical type specimens of *Pseudocassinarugosa* (ZMB-26915) and *Pseudocassinaocellata* (ZMB-26913). Bootstrap values are indicated at the nodes. https://binary.pensoft.net/file/766804This dataset is made available under the Open Database License (http://opendatacommons.org/licenses/odbl/1.0/). The Open Database License (ODbL) is a license agreement intended to allow users to freely share, modify, and use this Dataset while maintaining this same freedom for others, provided that the original source and author(s) are credited.Sandra Goutte, Jacobo Reyes-Velasco, Abeje Kassie, Stéphane Boissinot

Supplementary material 4 Aggressive call of *Leptopelisdiffidens* and aggressive or warm-up call of *Leptopelissuanae* Data type jpg file Explanation note **A** Aggressive call of *Leptopelisdiffidens* (SB128) and **B** aggressive or warm-up call of *Leptopelissuanae* (SB223). https://binary.pensoft.net/file/766805This dataset is made available under the Open Database License (http://opendatacommons.org/licenses/odbl/1.0/). The Open Database License (ODbL) is a license agreement intended to allow users to freely share, modify, and use this Dataset while maintaining this same freedom for others, provided that the original source and author(s) are credited.Sandra Goutte, Jacobo Reyes-Velasco, Abeje Kassie, Stéphane Boissinot

Supplementary material 5 Tables Data type localities, genetic, and morphometric data Explanation note **Tables: S1.** Localities of sampled specimens; **S2.** Number and average length of raw reads extracted from historical type specimens; **S3.** GenBank accession numbers; **S4.** PartitionFinder models; **S5.** Pairwise genetic distances (uncorrected P distances) of the whole mtDNA dataset; **S6.** airwise genetic distances (uncorrected P distances) of the COX1 dataset; **S7.** Linear morphometric measurements; **S8.** PCA loadings and scores for males and females of the *Leptopelisgramineus* complex; **S9.** Tukey HSD pairwise tests for linear morphometric measurements of males and females of the *Leptopelisgramineus* complex; **S10.** Acoustic characteristics of advertisement calls, averaged per individual. https://binary.pensoft.net/file/766806This dataset is made available under the Open Database License (http://opendatacommons.org/licenses/odbl/1.0/). The Open Database License (ODbL) is a license agreement intended to allow users to freely share, modify, and use this Dataset while maintaining this same freedom for others, provided that the original source and author(s) are credited.Sandra Goutte, Jacobo Reyes-Velasco, Abeje Kassie, Stéphane Boissinot

## References

[B1] Ahl E (1924) Über eine Froschsammlung aus Nordost-Afrika und Arabien. Mitteilungen aus dem Zoologischen Museum in Berlin 11: 1–12. 10.1002/mmnz.4830110102

[B2] Assefa E Bork H-R (2014) Deforestation and Forest Management in Southern Ethiopia: Investigations in the Chencha and Arbaminch Areas. Environmental Management 53(2): 284–299. 10.1007/s00267-013-0182-x 24292396

[B3] Boulenger GA (1896) LXX.—Descriptions of two new frogs from Lake Tanganyika, presented to the British Museum by Mr. W. H. Nutt. Annals & Magazine of Natural History 18(108): 467–468. 10.1080/00222939608680490

[B4] Boulenger GA (1898) Concluding report on the late Capt. Bottego’s collection of reptiles and batrachians from Somaliland and British East Africa. Annali del Museo Civico di Storia Naturale di Genova 2: 715–722.

[B5] Boulenger GA (1909) Descriptions of three new frogs discovered by Dr. P. Krefft in Usambara, German East Africa. Annals & Magazine of Natural History 4(24): 496–497. 10.1080/00222930908692705

[B6] Dabney J Meyer M Pääbo S (2013) Ancient DNA damage. Cold Spring Harbor Perspectives in Biology 5(7): a012567. 10.1101/cshperspect.a012567 PMC3685887 23729639

[B7] Dessie G Christiansson C (2008) Forest Decline and Its Causes in the South-Central Rift Valley of Ethiopia: Human Impact over a One Hundred Year Perspective. Ambio 37(4): 263–271. 10.1579/0044-7447(2008)37[263:FDAICI]2.0.CO;2 18686505

[B8] Freilich X Anadón JD Bukala J Calderon O Chakraborty R Boissinot S Calderon D Kanellopoulos A Knap E Marinos P Mudasir M Pirpinas S Rengifo R Slovak J Stauber A Tirado E Uquilas I Velasquez M Vera E Wilga A (2016) Comparative Phylogeography of Ethiopian anurans: Impact of the Great Rift Valley and Pleistocene climate change. BMC Evolutionary Biology 16(1): e206. 10.1186/s12862-016-0774-1 PMC5057412 27724843

[B9] Frost DR (2021) Amphibian Species of the World: an Online Reference. Version 6.1. https://amphibiansoftheworld.amnh.org/index.php [July 1, 2022]

[B10] Gordon A Hannon G (2010) FASTX-Toolkit: FASTQ/A short-reads preprocessing tools. http://hannonlab.cshl.edu/fastx_toolkit

[B11] Goutte S Reyes-Velasco J Boissinot S (2019) A new species of puddle frog from an unexplored mountain in southwestern Ethiopia (Anura, Phrynobatrachidae, *Phrynobatrachus*). ZooKeys 824: 53–70. 10.3897/zookeys.824.31570 PMC6381079 30799972

[B12] Goutte S Reyes-Velasco J Freilich X Kassie A Boissinot S (2021) Taxonomic revision of grass frogs (Ptychadenidae, *Ptychadena*) endemic to the Ethiopian highlands. ZooKeys 1016: 77–141. 10.3897/zookeys.1016.59699 33628080 PMC7892535

[B13] Hahn C Bachmann L Chevreux B (2013) Reconstructing mitochondrial genomes directly from genomic next-generation sequencing reads—A baiting and iterative mapping approach. Nucleic Acids Research 41(13): e129. 10.1093/nar/gkt371 PMC3711436 23661685

[B14] IUCN (2022) The IUCN red list of threatened species. https://www.iucnredlist.org [July 22, 2022]

[B15] IUCN Standards and Petitions Committee (2022) Guidelines for Using the IUCN Red List Categories and Criteria. Version 15.1. https://www.iucnredlist.org/documents/RedListGuidelines.pdf

[B16] Katoh K Standley DM (2013) MAFFT Multiple Sequence Alignment Software Version 7: Improvements in Performance and Usability. Molecular Biology and Evolution 30(4): 772–780. 10.1093/molbev/mst010 23329690 PMC3603318

[B17] Kindu M Schneider T Teketay D Knoke T (2015) Drivers of land use/land cover changes in Munessa-Shashemene landscape of the south-central highlands of Ethiopia. Environmental Monitoring and Assessment 187(7): e452. 10.1007/s10661-015-4671-7 26092242

[B18] Köhler J Jansen M Rodríguez A Kok PJR Toledo LF Emmrich M Glaw F Haddad CFB Rödel M-O Vences M (2017) The use of bioacoustics in anuran taxonomy: Theory, terminology, methods and recommendations for best practice. Zootaxa 4251(1): 1–124. 10.11646/zootaxa.4251.1.1 28609991

[B19] Koppetsch T (2020) A new species of *Trachylepis* (Squamata: Scincidae) from the Amhara Region, Ethiopia, and a key to the Ethiopian Trachylepis. Zootaxa 4859(1): 113–126. 10.11646/zootaxa.4859.1.4 33056207

[B20] Kostin DS Martynov AA Komarova VA Alexandrov DY Yihune M Kasso M Bryja J Lavrenchenko LA (2020) Rodents of Choke Mountain and surrounding areas (Ethiopia): The Blue Nile gorge as a strong biogeographic barrier. Journal of Vertebrate Biology 69(2): 1–12. 10.25225/jvb.20016

[B21] Kumar S Stecher G Li M Knyaz C Tamura K (2018) MEGA X: Molecular Evolutionary Genetics Analysis across Computing Platforms. Molecular Biology and Evolution 35(6): 1547–1549. 10.1093/molbev/msy096 29722887 PMC5967553

[B22] Lanfear R Calcott B Ho SYW Guindon S (2012) Partitionfinder: Combined selection of partitioning schemes and substitution models for phylogenetic analyses. Molecular Biology and Evolution 29(6): 1695–1701. 10.1093/molbev/mss020 22319168

[B23] Largen MJ (1977) The Status of the Genus *Leptopelis* (amphibia AnuraHyperoliidae) in Ethiopia, Including Descriptions of Two New Species. Monitore Zoologico Italiano. Supplemento 9: 85–136. 10.1080/03749444.1977.10736845

[B24] Largen MJ (2001) Catalogue of the amphibians of Ethiopia, including a key for their identification. Tropical Zoology 14(2): 307–402. 10.1080/03946975.2001.10531159

[B25] Lê S Josse J Husson F (2008) FactoMineR: An R package for multivariate analysis. Journal of Statistical Software 25(1): 1–18. 10.18637/jss.v025.i01

[B26] Lleonart J Salat J Torres GJ (2000) Removing Allometric Effects of Body Size in Morphological Analysis. Journal of Theoretical Biology 205(1): 85–93. 10.1006/jtbi.2000.2043 10860702

[B27] Manthey JD Reyes-Velasco J Freilich X Boissinot S (2017) Diversification in a biodiversity hotspot: Genomic variation in the river frog *Amietianutti* across the Ethiopian Highlands. Biological Journal of the Linnean Society. Linnean Society of London 122(4): 801–813. 10.1093/biolinnean/blx106

[B28] Mengistu AA (2012) Amphibian diversity, distribution and conservation in the Ethiopian highlands: morphological, molecular and biogeographic investigation on *Leptopelis* and *Ptychadena* (Anura), 219 pp.

[B29] Miller MA Pfeiffer W Schwartz T (2010) Creating the CIPRES Science Gateway for inference of large phylogenetic trees. In: 2010 Gateway Computing Environments Workshop (GCE), 1–8. 10.1109/GCE.2010.5676129

[B30] Neumann O (1902) From the Somali Coast through Southern Ethiopia to the Sudan. The Geographical Journal 20(4): 373–398. 10.2307/1775561

[B31] Onn CK Abraham RK Grismer JL Grismer LL (2018) Elevational size variation and two new species of torrent frogs from Peninsular Malaysia (Anura: Ranidae: *Amolops* Cope). Zootaxa 4434(2): 250. 10.11646/zootaxa.4434.2.2 30313185

[B32] R Core Team (2020) R: A language and environment for statistical computing. R Foundation for Statistical Computing, Vienna. http://www.R-project.org/

[B33] Reyes-Velasco J Manthey JD Freilich X Boissinot S (2018) Diversification of African tree frogs (genus *Leptopelis*) in the highlands of Ethiopia. Molecular Ecology 27(9): 2256–2270. 10.1111/mec.14573 29603468

[B34] Reyes-Velasco J Goutte S Freilich X Boissinot S (2021) Mitogenomics of historical type specimens clarifies the taxonomy of Ethiopian *Ptychadena* Boulenger, 1917 (Anura, Ptychadenidae). ZooKeys 1070: 135–149. 34819775 10.3897/zookeys.1070.66598 PMC8604866

[B35] Ronquist F Teslenko M van der Mark P Ayres DL Darling A Höhna S Larget B Liu L Suchard MA Huelsenbeck JP (2012) MrBayes 3.2: Efficient Bayesian Phylogenetic Inference and Model Choice across a Large Model Space. Systematic Biology 61(3): sys029. 10.1093/sysbio/sys029 PMC3329765 22357727

[B36] Rossing TD [Ed.] (2007) Springer Handbook of Acoustics. Springer New York, NY, XXIV, 1182 pp. https://link.springer.com/book/10.1007/978-0-387-30425-0 [July 17, 2022]

[B37] Shedlock AM Haygood MG Pietsch TW Bentzen P (1997) Enhanced DNA extraction and PCR amplification of mitochondrial genes from formalin-fixed museum specimens. BioTechniques 22(3): 394–396, 398, 400. 10.2144/97223bm03 9067006

[B38] Smith MA Poyarkov Jr NA Hebert PDN (2008) DNA BARCODING: CO1 DNA barcoding amphibians: take the chance, meet the challenge. Molecular Ecology Resources 8(2): 235–246. 10.1111/j.1471-8286.2007.01964.x 21585765

[B39] Soultan A Wikelski M Safi K (2020) Classifying biogeographic realms of the endemic fauna in the Afro-Arabian region. Ecology and Evolution 10(16): 8669–8680. 10.1002/ece3.6562 32884649 PMC7452816

[B40] Sprecht R (2017) Avisoft-SASlab Pro. Avisoft Bioacoustics, Berlin. http://avisoft.com/index.html

[B41] Sueur J Aubin T Simonis C (2008) Seewave, a free modular tool for sound analysis and synthesis. Bioacoustics-the International Journal of Animal Sound and Its Recording 18(2): 213–226. 10.1080/09524622.2008.9753600

[B42] Tiutenko A Zinenko O (2021) A new species of *Leptopelis* (Anura, Arthroleptidae) from the south-eastern slope of the Ethiopian Highlands, with notes on the *Leptopelisgramineus* species complex and the revalidation of a previously synonymised species. ZooKeys 1023: 119–150. 10.3897/zookeys.1023.53404 33776517 PMC7973069

[B43] Watters JL Cummings ST Flanagan RL Siler CD (2016) Review of morphometric measurements used in anuran species descriptions and recommendations for a standardized approach. Zootaxa 4072(4): 477–495. 10.11646/zootaxa.4072.4.6 27395941

