# Supplementary figures and images for: ﻿Genetic and morphometric analyses of historical type specimens clarify the taxonomy of the Ethiopian Leptopelisgramineus species complex (Anura, Arthroleptidae)
Source: Zookeys. 2022 Nov 8;1128:63–97. doi: 10.3897/zookeys.1128.82176 (PMC9836736; doi:10.3897/zookeys.1128.82176)

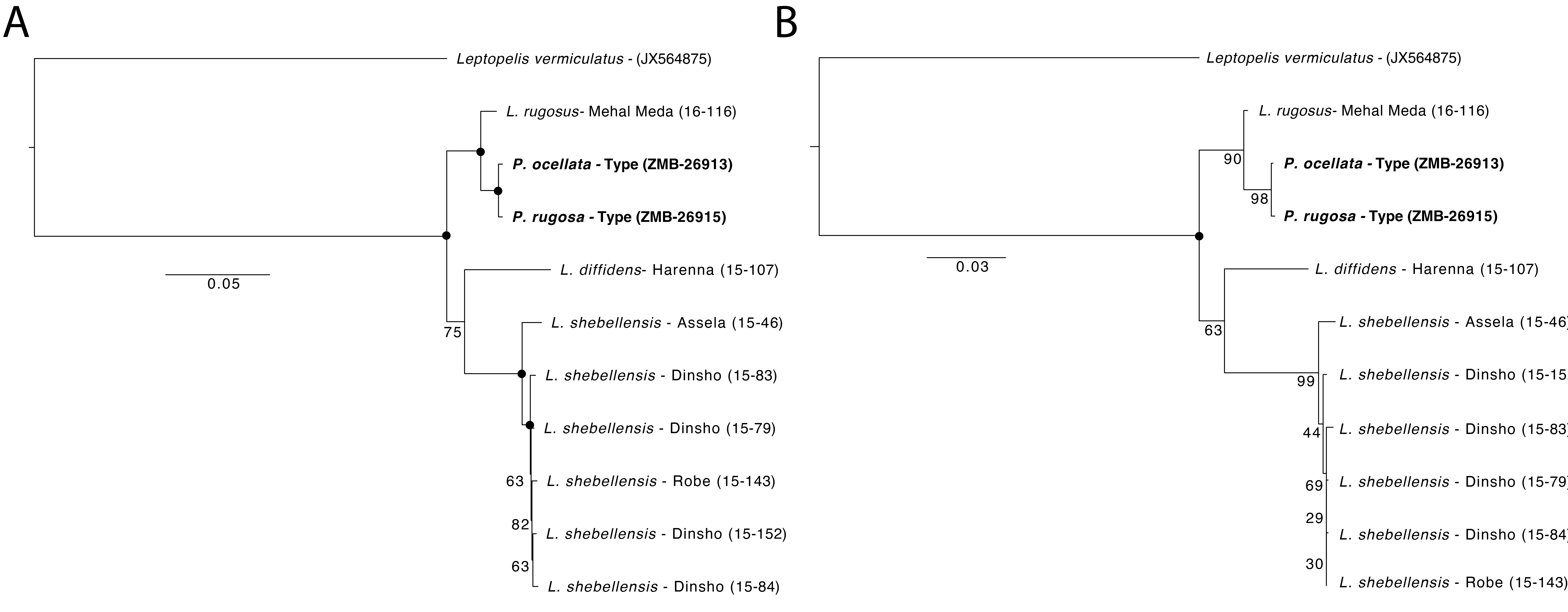

Supplement: Supplementary material 1 — Maximum Likelihood phylogenetic inference [file zookeys-1128-063_article-82176__-s001.jpg]

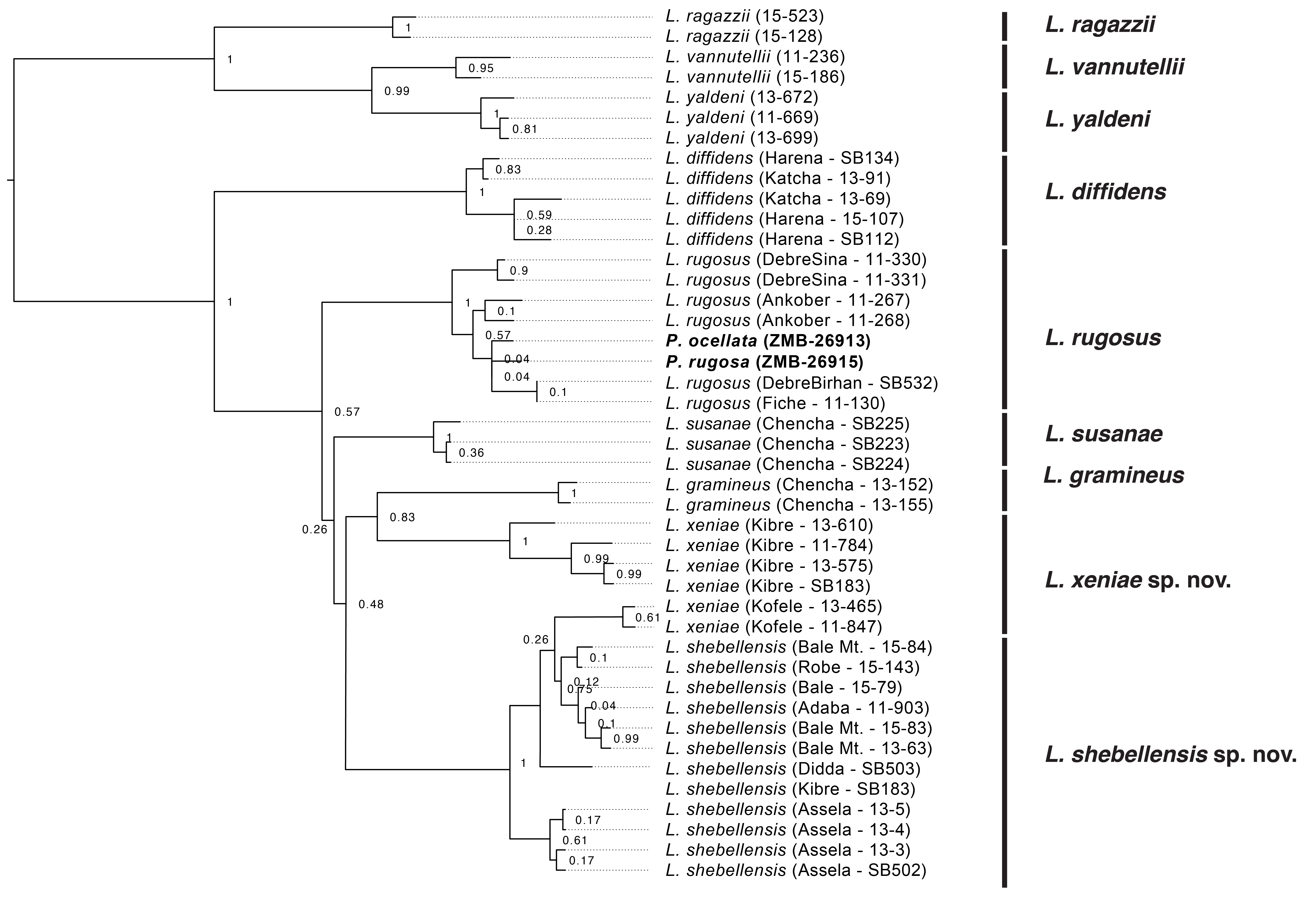

Supplement: Supplementary material 2 — Bayesian phylogenetic inference of the Leptopelisgramineus species complex, based on COX1 [file zookeys-1128-063_article-82176__-s002.jpg]

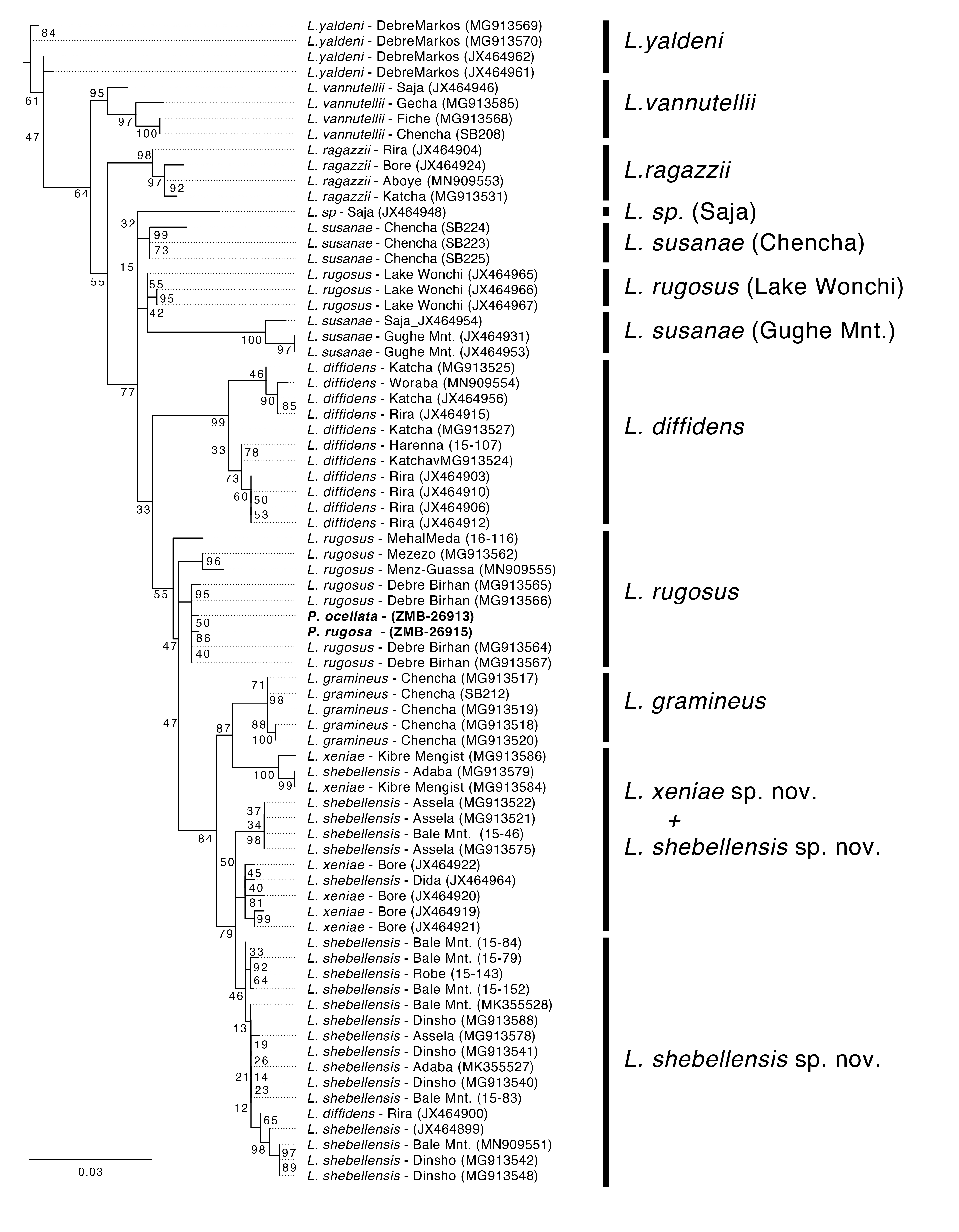

Supplement: Supplementary material 3 — Maximum Likelihood phylogenetic inference, based on the ribosomal RNAs 16s for members of the Leptopelisgramineus complex [file zookeys-1128-063_article-82176__-s003.jpg]
